# Cortical folding correlates to aging and Alzheimer’s Disease’s cognitive and CSF biomarkers

**DOI:** 10.1038/s41598-023-50780-3

**Published:** 2024-02-08

**Authors:** Fernanda Hansen P. de Moraes, Felipe Sudo, Marina Carneiro Monteiro, Bruno R. P. de Melo, Paulo Mattos, Bruno Mota, Fernanda Tovar-Moll

**Affiliations:** 1 Brain Connectivity Unit, D’Or Institute of Research and Education (IDOR), Rio de Janeiro, 225281-100 Brazil; 2 Instituto de Física, Universidade Federal do Rio de Janeiro (UFRJ), Rio de Janeiro, 21941-909 Brazil; 3 Memory Clinic, D’Or Institute of Research and Education (IDOR), Rio de Janeiro, 225281-100 Brazil

**Keywords:** Diagnostic markers, Alzheimer's disease, Biophysical models

## Abstract

This manuscript presents the quantification and correlation of three aspects of Alzheimer’s Disease evolution, including structural, biochemical, and cognitive assessments. We aimed to test a novel structural biomarker for neurodegeneration based on a cortical folding model for mammals. Our central hypothesis is that the cortical folding variable, representative of axonal tension in white matter, is an optimal discriminator of pathological aging and correlates with altered loadings in Cerebrospinal Fluid samples and a decline in cognition and memory. We extracted morphological features from T1w 3T MRI acquisitions using FreeSurfer from 77 Healthy Controls (age = 66 ± 8.4, 69% females), 31 Mild Cognitive Impairment (age = 72 ± 4.8, 61% females), and 13 Alzheimer’s Disease patients (age = 77 ± 6.1, 62% females) of recruited volunteers in Brazil to test its discriminative power using optimal cut-point analysis. Cortical folding distinguishes the groups with reasonable accuracy (Healthy Control-Alzheimer’s Disease, accuracy = 0.82; Healthy Control-Mild Cognitive Impairment, accuracy = 0.56). Moreover, Cerebrospinal Fluid biomarkers (total Tau, A$\beta$1-40, A$\beta$1-42, and Lipoxin) and cognitive scores (Cognitive Index, Rey’s Auditory Verbal Learning Test, Trail Making Test, Digit Span Backward) were correlated with the global neurodegeneration in MRI aiming to describe health, disease, and the transition between the two states using morphology.

## Introduction

Alzheimer’s Disease is the most common dementia worldwide. The clinical diagnosis of Alzheimer’s Disease is not based on morphometric variables: it relies on episodic memory impairment, neuropsychological assessment, at least one abnormal biomarker among Cerebrospinal fluid (CSF) analysis, and neuroimaging (PET and MRI)^1^. During its prodromal stage, Mild Cognitive Impairment, some cognitive dysfunction is present but to a lesser extent than in dementia. The Mild Cognitive Impairment condition is, therefore, non-determinant of future conversion to dementia and can be heterogeneous depending on which symptoms are present and if the patient ever converts^2^. Alzheimer’s Disease is also characterized by altered concentration of Amyloid $\beta$ (A$\beta$) 1-40, A$\beta$1-42, and total Tau (t-Tau) protein on the CSF, which is correlated with findings of amyloid plaques and Tau tangles on histopathological examinations^3,4^. In addition, new biomarkers for Alzheimer’s Disease have been suggested based on the pathology’s inflammation, such as Lipoxin, which regulates chronic inflammatory process resolution^5^. In structural images, Alzheimer’s Disease is characterized by brain atrophy, which includes volume reductions in the medial temporal lobe and hippocampus, grey matter loss with consequent reduced cortical thickness^6–8^.

In recent years, cortical folding quantification has been proposed as a promising new approach to correlate morphological measurements to healthy aging^9–12^ and alterations in brain structure due to neurological pathologies, including Alzheimer’s Disease^13–15^. Cortical folding can be measured from a structural T1-weighted MRI: (i) by using the primary parameter of cortical folding, Gyrification Index (GI), the ratio of the grey matter’s Total and its Exposed Areas^16^, (ii) by calculating the fractal dimension^17^ of the cortical surface, and (iii) by calculating an index (k) derived from a power-law relationship of Total Area ($A_T$), Exposed Areas ($A_E$), and Cortical Thickness (T). The latter was proposed as a physics-based model for cortical folding by Mota & Herculano-Houzel and validated for 55 mammals^18^. The model provides two indexes of cortical folding: its linear coefficient (k), a natural variable to describe brain morphology, which by theory represents the axonal tension, and the angular coefficient ($\alpha$), a constant with self-similarity properties and theoretical value of 1.25.

Wang and colleagues further described the cortical folding universal rule from Mota & Herculano-Houzel for the human brain across gender, age, and pathology^19,20^. The model evolved to a vector base space that describes the mammals’ cortex in three dimensions: axonal tension, K (Eq. 1), cortical shape complexity S (Eq. 2), and the brain isometric volume I (Eq. 3). The original work proposes those three perpendicular, independent, and dimensionless variables combined could help distinguish pathological events similar to age effects, such as Alzheimer’s Disease^21^.

1 $$\begin{aligned} \text {K}&=  \log _{10}{\text {k}}~=~\log _{10}{\text {A}_\text {T}}-\frac{5}{4}\log _{10}{\text {A}_\text {E}}+\frac{1}{4}\log _{10}{\text {T}^2} \end{aligned}$$

2 $$\begin{aligned} S&= \frac{3}{2}\log _{10}{{\text {A}}_{\text {T}}}-\frac{3}{4}\log _{10}{{\text {A}}_{\text {E}}}-\frac{9}{4}\log _{10}{{\text {T}}^2} \end{aligned}$$

3 $$\begin{aligned} I~&=  ~\log _{10}{{\text {A}}_{\text {T}}}+\log _{10}{{\text {A}}_{\text {E}}}+\log _{10}{{\text {T}}^2} \end{aligned}$$

We propose improving the differential diagnosis of Alzheimer’s Disease, Mild Cognitive Impairment, and Healthy Controls by using the least variant component of the proposed new vector base and representative of axonal tension, K^22^. Lastly, we correlate K, representing pathological structural changes, with neuropsychological tests used to diagnose dementia (cognitive function, working and episodic memory, and memory estimation), typical CSF biomarkers related to Alzheimer’s Disease (t-Tau, A$\beta$1-40, A$\beta$1-42), and Lipoxin.

## Results

**Figure 1:**
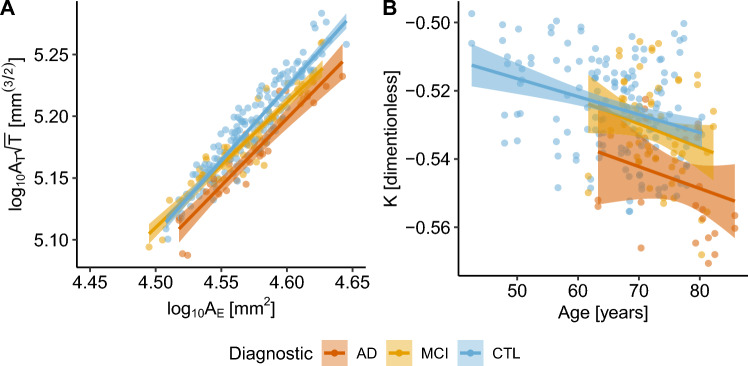
Age and diagnostic effects in cortical gyrification. (**A**) Linear fitting with 95% Confidence Interval (CI) for the model variables in each Diagnostic group, Healthy Controls (CTL, in blue, adjusted R^2^ = 0.85, *P* < 0.0001), Mild Cognitive Impairment (MCI, in yellow, adjusted R^2^ = 0.88, *P* < 0.0001), and Alzheimer’s Disease (AD, in red, adjusted R^2^ = 0.86, *P* < 0.0001). As the severity of the disease increases, the linear tendency is downshifted, with smaller linear intercepts (K). (**B**) K linear tendency across age with 95% CI for the three diagnostics groups: Alzheimer’s Disease (in red, adjusted R^2^ = 0.026, *P* = 0.21), Mild Cognitive Impairment (in yellow, adjusted R^2^ = 0.044, *P* = 0.0051), and Healthy Controls (in blue, adjusted R^2^ = 0.097, *P* < 0.0001).

Firstly, we verified if the IDOR dataset fit the universal cortical folding model proposed by Mota and Herculano-Houzel^18^ by estimating the linear coefficient $\alpha$ and quantifying the model’s linearity. The IDOR data fits the proposed linear model with $\alpha$ = 1.14±0.03 [1.07, 1.20] (R^2^ = 0.83, *P* < 0.001) statistically different from the theoretical value, 1.25 (Student’s t = 3.38, *P* = 0.00084). Further, we verified if the proposed cortical folding variables from the Mota & Herculano-Houzel model^18^ and the derived variable K^21^ correlates with aging. Healthy aging reduces brain gyrification in terms of self-similarity $\alpha$ (Pearson’s r = -0.79, DF = 5, *P* = 0.0345, d = -2.58 [-6.62, 1.47]) (Supplementary Note 2) and axonal tension K (Pearson’s r = -0.32, DF = 152, *P* < 0.0001, d = -0.68 [-1.15, -0.2]) (Fig. 1).

### Diagnostic discrimination

In terms of Diagnostic groups discrimination using cortical folding variable K related to axonal tension, K is different for the diagnostic groups in the hemisphere (ANOVA F = 28.51, DF = 2, *P* < 0.0001, *P* < 0.01 for all pairwise comparisons), meaning a global structural change with the pathology. The decrease of K with disease presents a similar pattern of the decline with healthy aging, in which Cortical Thickness, Exposed, and Total Areas are reduced, promoting a reduction in cortical folding.

Considering specific lobes, Alzheimer’s Disease, Mild Cognitive Impairment, and Healthy Controls presented differences in gyrification in all lobes (Frontal lobe, F = 15.04, DF = 2, *P* < 0.0001; Occipital lobe, F = 10.1, DF = 2, *P* < 0.0001; Parietal lobe, F = 16.82, DF = 2, *P* < 0.0001 and Temporal lobe, F = 27.61, DF = 2, *P* < 0.0001). Subsequent pairwise comparisons showed significant differences between Healthy Controls-Alzheimer’s Disease and Mild Cognitive Impairment-Alzheimer’s Disease for all lobes and a significant difference between Healthy Controls and Mild Cognitive Impairment for the Temporal lobe (Supplementary Information, Fig. S2). There is no statistical power to infer if the difference in K between Alzheimer’s Disease and Healthy Controls increases with age (Supplementary Information, Fig. S3).

**Figure 2:**
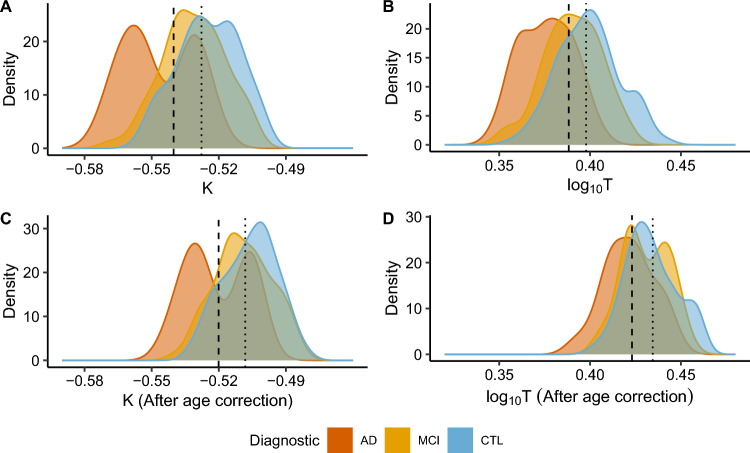
Optimal cut-point (maximum sensitivity and specificity) for K and Cortical Thickness, including results with removed age effect (“age correction”). The dashed line represents the optimal cut-point to discriminate Alzheimer’s Disease (AD, in red) and Healthy Controls (CTL, in blue), and the dotted line represents the optimal cut-point for Mild Cognitive Impairment (MCI, in yellow) and Healthy Controls. (**A**) For K, the optimal cut-point for the CTL-AD is -0.54, and for CTL-MCI, -0.53. (**B**) For $log_{10}{T}$, the optimal cut-point for CTL-AD = 0.39 mm and CTL-MCI = 0.40 mm. (**C**) For K, after age correction, the optimal cut-point for CTL-AD = -0.52 and CTL-MCI = -0.51. (**D**) For $log_{10}{T}$, after age correction, the optimal cut-point for CTL-AD = 0.43 mm and Healthy Controls-Mild Cognitive Impairment = 0.44 mm.

We evaluated K and Cortical Thickness ($log_{10}{T}$) optimal cut-points in raw data and after removing the age effect to compare their discriminating power (Fig. 2). K optimal cut-point for discriminating Alzheimer’s Disease and Cognitive Unimpaired Controls is -0.54, and for discriminating Mild Cognitive Impairment and Controls, -0.53. K has excellent accuracy and reasonable specificity in distinguishing Alzheimer’s Disease from Healthy Controls (AUC = 0.84, accuracy = 0.82, specificity = 0.86, ROC curve in Supplementary Information Fig. S4), and low sensitivity (0.58), while $log_{10}{T}$ (cut-point = 0.39, AUC = 0.85, accuracy = 0.73) has a balanced trade-off with specificity and sensitivity (0.77 and 0.73 respectively). Discriminating Mild Cognitive Impairment from Healthy Controls is challenging for both K (AUC = 0.63, accuracy = 0.56, specificity = 0.83, sensitivity = 0.54, ROC curve in Supplementary Information Fig. S4) and $log_{10}{T}$ (AUC = 0.64, accuracy = 0.57, specificity = 0.44, sensitivity = 0.58).

K (after age correction) cut-points for lobes are described in Table 1 (expanded results in Supplementary Information Table S1). It is possible to verify that local cortical regions are less prone to diagnostic discrimination than the whole cortex. However, we highlight the discrimination of Healthy Controls and Alzheimer’s Disease (AD) patients in the parietal lobe (AUC = 0.82, accuracy = 0.75) and in the temporal lobe for the Healthy Controls (CTL) and Mild Cognitive Impairment (MCI) group contrast (AUC = 0.67, accuracy = 0.59).

**Table 1:** Optimal cut-points (maximum sensitivity + specificity) for K (age-corrected) at each lobe (Frontal, F; Occipital, O; Parietal, P, and Temporal, T). Cut-points, accuracy, sensibility, and specificity for discriminating pairwise diagnostic groups.

Lobes	CTL-AD					CTL-MCI				
	Cut-point	AUC	ACC	SENS	SPEC	Cut-point	AUC	ACC	SENS	SPEC
F	− 0.46	0.70	0.73	0.46	0.78	− 0.45	0.55	0.5	0.54	0.48
O	− 0.35	0.67	0.73	0.5	0.77	− 0.33	0.56	0.45	0.79	0.32
P	− 0.34	0.79	0.72	0.77	0.72	− 0.34	0.47	0.64	0.30	0.77
T	− 0.34	0.75	0.67	0.61	0.68	− 0.34	0.59	0.69	0.41	0.80

*AUC* area under the curve, *ACC* accuracy, *SENS* sensibility, and *SPEC* Specificity. ROI codes: F, frontal; O, occipital; P, parietal, and T, temporal lobes.

Potential confounders to account for with the available data in the diagnostic discrimination analyses are age, sex, and years of Education. Age, as demonstrated, has significative effects on K and is corrected by removing the age effect. Gender and Education have significant but small effects on K and therefore were not treated as confounders in these results. Gender accounts for 2.6% of K variance and 3.9% of K (age corrected) variance. Education accounts for 0.70% and 0.42% of K and K (age corrected) variances. Extended results and a brief discussion are in Supplementary Note 4.

The validation of these results with a subsample of the ADNI dataset (Supplementary Note 5) presented lower AUC and accuracy for K and $log_{10}{T}$. The cut-point values cannot be compared directly due to inherent systematic variance in acquisition and processing.

### Morphological correlation between neurodegeneration and Behavioral and CSF assessments

Further, we aimed to verify if the Alzheimer’s Disease-related morphological brain changes would be correlated to clinical and behavioral variables and CSF biomarkers of the disease. We found significant correlations between a) executive function, either measured by Digit Span Backwards (working memory) or TMT B-A (cognitive flexibility), b) memory measured by RAVLT A7/A5, and c) global cognition measured by the Cognitive Index with K and Cortical Thickness (Table 2 and extended results in Supplementary Information Table S2). The severity of cognitive symptoms was associated with decreased cortical folding and decreased Cortical Thickness.

**Table 2:** Pearson Correlations (r) and Effect Size (d) for behavioral and biochemical assessments and morphological parameters, K and Cortical Thickness ($log_{10}{T}$).

Clinical Assessment	ROI	K [r; d]	$log_{10}{T}$ [r; d]
Behavioral			
Cognitive Index	H	0.41*; 0.9	0.409*; 0.87
RAVLT A7/A5	H	0.36*; 0.77	0.39*; 0.85
	T	0.31*; 0.65	0.44*; 0.98
TMT B-A	H	− 0.30*; − 0.63	− 0.22*; − 0.45
	F	− 0.21*; − 0.43	− 0.21*; − 0.43
Digit Span Backward	H	0.25*; 0.52	0.20*; 0.41
	F	0.21*; 0.43	0.21*; 0.43
Biochemical (CSF)			
A$\beta$1-40 [pg/mL]	H	− 0.073; − 0.15	− 0.22*; − 0.45
A$\beta$1-42 [pg/mL]	H	0.26*; 0.54	0.086; 0.17
t-Tau [pg/mL]	H	− 0.26*; − 0.54	− 0.41*; − 0.9
A$\beta$1-42/ A$\beta$1-40	H	0.18; 0.37	0.20; 0.41
t-Tau/A$\beta$1-42	H	− 0.32*; − 0.68	− 0.34*; − 0.72
t-Tau/(A$\beta$1-42/ A$\beta$1-40)	H	− 0.28*; − 0.58	− 0.34*; − 0.72
Lipoxin [pg/mL]	H	0.11; 0.22	− 0.052; − 0.10

The *P* value was corrected (Bonferroni) for multiple comparisons within Clinical Assessment and morphological measurement. ROI codes: H, Hemisphere; F, Frontal Lobe, and T, Temporal Lobe.

We found significant correlations between K and the concentration of t-Tau, A$\beta$1-42, and ratios of Tau and A$\beta$ concentrations. Decreased cortical folding (lower K) was associated with elevated concentrations of t-Tau and its ratios with A$\beta$ and reduced concentrations of A$\beta$1-42 in CSF.

## Discussion

Structural MRI imaging biomarkers have been extensively studied, including cortical folding aspects of Alzheimer’s Disease^23^ and Mild Cognitive Impairment^24^. However, investigating cortical morphological measurements (or their combinations) is not straightforward since only a few parameters will lead to biological interpretations and adequate characterizations of the event in the study. Inspired by the cortical folding model proposed by Mota & Herculano-Houzel^18^, we have investigated an improved and well-motivated structural biomarker to better discriminate between diseased and healthy aged brains. The biomarker derived from the cortical folding model is the variable K, characterized by a biomechanical interpretation with very low variance across species and healthy adult humans^19^. We have shown that K discriminates patients with Alzheimer’s Disease from Mild Cognitive Impairment and age-matched controls. Further, we have demonstrated that structural damages described by K correlate with cognitive decline and biochemical CSF changes related to Alzheimer’s Disease.

From the chosen independent parameters, K (or $log_{10}{k}$) is a natural descriptor of cortical folding and global brain morphology as it is: i) almost invariant across mammals, including lissencephalic species and cetaceans; ii) derived from physical principles, iii) aggregate structural information from Areas and Cortical Thickness, and iv) is based on empirical evidence. With the development of this cortical folding model, there was an addition of the auxiliary, but not as invariant within Healthy Controls as K, variables S and I that are defined to be independent of K and can carry information about either shape complexity or size exclusively^21^.

It is important to note, however, that the model proposed in Mota & Herculano-Houzel^18^, from which both the theoretical scaling law and the new morphometric variables were derived, is not the only proposed mechanism for cortical gyrification. This is a simple model that addresses important aspects but does not explain all the features known to affect gyrified cortices, such as systematic variations in cortical thickness, buckling induced by differential rates of lateral expansion^25^, the prevalence of U-shaped fiber between gyri, the presence of cortical-thalamic connections or any role for other sub-cortical structures. This simplicity is, at the same time, the model’s greatest strength and weakness. On one hand, this means it cannot provide a complete description of cortical gyrification; on the other hand, it is general enough to predict the existence of a universal scaling law that was empirically verified across species, individuals, cortical regions, and length scales^18–21^. Indeed, this scaling law can be regarded as a strict and non-trivial test for any other proposed mechanism of cortical gyrification. To our knowledge, so far, no other proposed gyrification model has been shown to conform to it. More specifically, in this present work, the theoretical model plays the role of providing the scaling law to test new datasets against, enabling us to characterize normal and abnormal gyrification.

Corroborating the primary hypothesis in this study, our results suggest that K is a sensitive variable for differentiating between Alzheimer’s Disease, Mild Cognitive Impairment, and healthy aging while aggregating information about complex biological and theoretical processes. Specifically, for Alzheimer’s Disease applications, cortical folding variables, such as K, could become alternative candidate neurodegeneration biomarker in the NIA-AA AT(N) framework^26^ and its updated version, the ATX(N) framework if a more extensive cut-point analysis is made to ensure its flexibility across multiple datasets and its diagnostic prediction power^27^. In this study, the subject’s assessment covered multiple clinical and behavioral domains and investigated biochemical CSF biomarkers of neurodegeneration, confirming that alteration in cortical gyrification (in K) correlates to changes in cognitive function and biochemical markers in Alzheimer’s Disease pathology. To investigate the hypothesis properly, we first verified if the model proposed by Mota & Herculano-Houzel^18^ was adequately fitted to our dataset. We verified the linear trend and compared $\alpha$ for each group with the theoretical value of 1.25 to confirm that our data fit the proposed model for gyrification. Then, K values were calculated to study how cortical gyrification changes in dementia, allowing comparisons with previous investigations.

The presented data fit the universal cortical folding power-law model with a lower value of $\alpha$ than the theoretical value and has a slope comparable to previous findings^19,20^. However, there are limitations in comparing our results to prior publications due to the differences in acquisition parameters, equipment, and FreeSurfer versions that could imply confounding components^28,29^. When comparing our data with a subset of the Amsterdam Open MRI Collection, AOMIC PIOP01^30^, acquired with the same scanner of IDOR data, the results suggest that the slope’s variation is mainly related to age. Thus, our supposed abnormal reported smaller $\alpha$ is probably due to the elder and diseased subjects present in the data, indicating a continuous loss in the brain self-similarity aspect. As an extrapolation of this result in concern to the folding theory proposed in the original report^18^ and considering $\alpha$ as a self-similarity index, we hypothesize the nonhomogeneity in the cerebral cortex (gray matter) due to aging and Alzheimer’s Disease leads to a non-fractal shape, here seen as a deviation from the theoretical constant with self-similarity properties (slope smaller than the theoretical value of 1.25). We expect expansions of this study to include the non-homogeneity in cortical structure in the theoretical model. We cross-validated these results with the Amsterdam Ultra-high field adult lifespan database (AHEAD), a 7T MRI structural images^31^ (Supplementary Note 3). These results confirm the previously reported dependency of gyrification with age globally and locally^32^.

To compare K with a known structural biomarker, Cortical Thickness (T), we estimated their optimal cut-points and relative Area Under the Curve (AUC), accuracy, specificity, and sensibility. Lobes’ cut-points were also determined. Our results suggest that an independent cortical morphology component, K, is of great potential utility, even when applied to smaller ROI, in agreement with previously reported results^33^. After removing the age effect to isolate the pathological effects on brain structure, we evaluated the optimal cut-point for Cortical Thickness and K. K still had higher accuracy than Cortical Thickness in discriminating between Healthy Controls and Alzheimer’s Disease and Healthy Controls and Mild Cognitive Impairment. We expanded this analysis by applying the same methodology to a subsample of the ADNI dataset processed and shared by Wang et al.^19^. We found different cut-points, which was expected due to the differences in socio-economic-demographics stats, selection bias, and image acquisition and processing methodology between the two samples (IDOR and ADNI) that could imply differences in morphological measurements, though the model accuracy is comparable.

Considering K is a neurodegeneration biomarker in Alzheimer’s Disease, the bi-modal shape of the curve indicates two influential groups with different levels of structural injuries, a distinction not detected by Cortical Thickness (bimodal distribution discussed in more detail in Supplementary Note 6). It is possible to affirm with a visual inspection of Fig. 2 that K is more sensitive to discriminate subjects with a less folded brain, probably due to Alzheimer’s Disease, more aggressive or distributed spatially pathological injuries. Future works should quantify the relation between global K and spatial distribution of brain atrophy, which is also an indicator of the disease’s later stages, confirming this interpretation^34^. The reduced number of subjects limits the statistical power of including subgroups in the cut-point analysis.

The accuracy is smaller when comparing Healthy Controls and Mild Cognitive Impairment, which is expected since the diagnostic includes a broad range of pathological involvement and outcome, not accounted for in this study due to the cross-sectional design. Mild Cognitive Impairment does not necessarily lead to a transition to any dementia in all cases, let alone to Alzheimer’s Disease. Besides, memory loss and reduced cognitive abilities are also present in healthy aging. The morphological characteristics of the Amnestic Mild Cognitive Impairment sample suggest that, as in the clinical aspects, the diagnostic works as an intermediate step. However, longitudinal expanded studies will be necessary to address this specific aspect.

Local analysis in lobes indicates K (after age correction) has better accuracy in discriminating between Healthy Controls and Alzheimer’s Disease in the Frontal and Parietal lobes, despite its very low sensibility. Higher sensibility is found in the Parietal lobe and higher specificity in the Frontal lobe. This finding confirms that the parietal lobes are affected by extended brain atrophy after the temporal lobes^20^. The higher accuracy and AUC in discriminating Mild Cognitive Impairment subjects from the Healthy Controls in the Temporal lobe further confirm that this region is the earlier spatial stage of brain atrophy. The significant difference between the discriminative power of K’s values with and without the age effect suggests that the parietal lobe is more affected by disease than aging.

It is not possible to confirm with this sample the results from Wang and colleagues^20^, if K is better at discriminating Alzheimer’s Disease from healthy aging in younger individuals (Supplementary Information, Fig. S3). An extension of this work would benefit from an increase in the number of subjects to further investigate this hypothesis.

As previous publications report, Alzheimer’s Disease and healthy aging have different biological bases and onset locations of degeneration that construct both cognitive degeneration processes, from hippocampal neuronal loss^35^ to the degradation of cognitive networks^36^. However, in morphological terms, Alzheimer’s Disease degeneration is similar to a premature and accelerated aging process. Our global and local analysis corroborates this indication as K is depreciated with aging for all diagnostics. In Wang et al., 2021^21^, it is proposed that including S and I would improve our knowledge about brain morphology and our capability to remove aging effects. Alzheimer’s Disease visually mimics K, S, and I aging in our sample and at ADNI (Supplementary Note 5).

Besides Cortical Thickness being one of the most studied morphological parameters for Alzheimer’s Disease, further studies should consider that K represents a natural variable that translates global and local changes in brain structure and is more sensitive to changes in the disease severity. In advancing past the present work, one might consider increasing the complexity in the discrimination model since none of the included morphological parameters in this analysis, cortical folding and cortical thickness delivered results that could be used in a clinical approach due to its low sensitivity and specificity in the smaller regions of interest. Further, the short- and medium-term variation in cortical folding and cortical thickness during the continuum of dementia should be deeply verified by a longitudinal study. Regarding specific brain locations affected by dementia, another challenge found by this study was to relate the global structural findings to alterations in subcortical structures such as the hippocampus, which is highly impacted by Alzheimer’s Disease in its later stages^37^.

Previous studies suggested the morphological alterations in a brain with Alzheimer’s Disease are not concurrent with biochemical and behavioral alterations in the pathological course^38^, which indicates abnormalities in A$\beta$ and t-Tau concentrations and brain morphology alterations precede the clinical symptoms. Moreover, one limitation of behavioral assessments is that they can include multiple cognitive domains and most complex tasks are not mapped in a single region of the human brain. For example, episodic memory decline can be related to the reduced number of neurons, synaptic efficiency, and the concentration of neurotransmitters, affecting the prefrontal cortex, medial temporal lobe, parietal cortex, and cerebellum successively^39^.

K and Cortical Thickness presented correlations for clinical scores, highlighting the Cognitive Index and the episodic memory score (RAVLT A7/A5). An expected correlation was also found for t-Tau/A$\beta$1-42 and t-Tau/(A$\beta$1-42/A$\beta$1-40), commonly used ratios to describe Alzheimer’s Disease effects. Therefore, regardless of the Diagnostic, the brain unfolds (measured by the decrease of variable K) with a smaller cognitive index, episodic memory score, auditory (Digit Span Backward), and visual working memory (Trail Making Test). In terms of the biochemical data analyzed, we can confirm that a less folded brain tends to have a higher concentration in CSF of t-Tau, t-Tau/A$\beta$1-42, and t-Tau/(A$\beta$1-42/A$\beta$140) ratios, and a lower concentration of A$\beta$1-42, probably, at least in part, due to its presence in the plaques^40^. These findings are expected as the onset of brain structure injuries is later than that of A$\beta$ plaques and phosphorylated Tau occurrence, leading to neuronal death^38^. We correlated K and the clinical/biochemical scores independently from the diagnostic, given that these events are not simultaneous in the pathological course.

We do not yet fully understand the contributions of deviation from biochemical and clinical typical values to the structural changes in dementia and neurodegenerative diseases. However, we can provide a time-point analysis and correlate the accumulation of A$\beta$ plaques and Tau tangles with reduced cortical thickness and a less folded brain. Also, previous reports describe gyrification changes in smaller regional ROIs associated with one domain tasks and cognitive index as Mini-Mental State Examination (MMSE)^41^. Núñez investigated the association between gyrification and memory scores in Alzheimer’s Disease subjects and reported significant associations between a semantic fluency test and the left insular cortex^42^. In contrast, we use a global measure of gyrification that is theoretically motivated and shown to be correlated to multiple cognitive measurements. Concerning the cortical folding variables, future studies must overcome the methodological limitations of comparing samples acquired on different sites to focus on the impact of socio-economic-demographic^43^, cognitive reserve^44^, and cognitive protection^45^ on K, S, and I.

We intended to minimize the limitation due to the restricted sample size used in this study by re-running the morphometrical analysis in a subsample of ADNI, included in Wang et al.^19^. However, in this methodological replication, there are limitations regarding the data and methodology to extract morphological variables. In the subsample included in Wang et al. work, there are no Mild Cognitive Impairment subjects. Also, most subjects do not present information about biospecimen collection and clinical data assessments. Inherent differences in acquisition and processing pipelines might lead to differences in morphological measurements which is the possible cause for finding different cut-points in the replication; therefore, validating the cut-points found from IDOR by applying it to ADNI data would not be effective as comparing the discriminative power of K in both situations. Nevertheless, we intended to suggest a clinical application of a novel morphological variable for measuring neurodegeneration, demonstrate its usefulness, and increase data diversity by studying a sample with recruited volunteers in a middle-income country and acknowledge the possible limitations of extending these results to other datasets.

Lastly, Alzheimer’s Disease is a complex pathology related to multiple lifestyle and social factors and health overcome^46^. We expect future studies to include other potential confounders that assess the disorder’s inherent complexity. Further, extensions of this study could verify the validity of this diagnostic tool in smaller regions of interest, such as specific regions, sulci, or gyri, as the proposed method suggested in work by Leiberg et al.^47^.

This manuscript intended to verify the clinical application of the proposed independent morphological components on 123 elder subjects and argues that a variable related to axonal tension and the invariant aspects of cortical gyrification should be considered an additional highly discriminating structural marker to describe neurodegeneration. As the biological meaning of K and S are confirmed in future studies, we expect to be able to infer the biomechanical process occurring in neurodegeneration, especially Alzheimer’s Disease, using independent morphological variables that we have proposed recently that better capture the global aspects of cortical shape and size^21^. We have shown that Alzheimer’s Disease is morphologically similar to accelerated aging and distinguishable from the Mild Cognitive Impairment and Healthy Controls groups. Further, we demonstrated significant correlations between K and multiple behavioral tests and CSF biomarkers, which are sensitive to age correction, reinforcing that non-concomitant processes during Alzheimer’s Disease make it harder to establish chains of causality.

## Methods

The Alzheimer’s project sustained by IDOR is a follow-up study about Alzheimer’s Disease’s morphological, behavioral, and biochemical aspects. The study enrolled 231 individuals from 2011 to 2018. Eligibility criteria for the project were as follows: (i) subjects had no contraindications to undergo MRI, such as presenting metal implants in the head; (ii) participants showed no signs or symptoms indicative of large-vessel cerebrovascular disease, tumoral changes, or traumatic injury affecting brain structure, as detected in clinical, cognitive and neuroimaging assessments; (iii) no severe sensorial deficits which could interfere in the application of neuropsychological tests were identified; (iv) subjects did not present major depressive disorder or any severe lifetime psychiatric disorder and (v) MRI analyses showed no significant artifacts, which could preclude the identification of brain structures. Also, subjects presenting anxiety or any other condition which interfered with their ability to remain still during MRI were excluded. Details on eligibility and exclusion criteria are described by Sudo et al.^48^. The Hospital Copa D’Or Research Ethics Committee approved the present research under protocol number CAAE 47163715.0.0000.5249. All the participants provided written informed consent before enrolment in the study and all experiments were performed in accordance with relevant guidelines and regulations. All images and data were anonymized after acquisition. In this study, we included a convenience sample by selecting the MRI first session and clinical assessment of 134 participants who met the inclusion criteria: Healthy Unimpaired Controls, or with the exclusive diagnosis of Mild Cognitive Impairment or Alzheimer’s Disease, and T1w structural MRI images acquired with the same protocol at the same equipment, a 3 T Philips Achieva, and eight years or more of Education (Flowchart at Supplementary Information Fig. S1, which include further details of exclusion in the data processing steps). The diagnosis was defined based on the criteria described in the Diagnostic and Statistical Manual of Mental Disorders (DSM-5)^49^.

### Data acquisition and processing

The participants’ T1-weighted MRI images (3 T Philips Achieva) were acquired with the following acquisition protocol: TR/TE 7.2/3.4 ms; matrix 240x240 mm; FOV 240 mm; slice thickness 1 mm; 170 slices. The structural images were processed in FreeSurfer v6.0.0 with the longitudinal pipeline (due to the multiple acquisitions for the cohort project, despite selecting only the first session) without manual intervention at the surfaces^50–52^ and with the localGI pipeline to generate an external surface^16^. Morphological measurements from the surfaces generated with FreeSurfer were extracted with Cortical Folding Analysis Tool^53^. We defined ROI as the whole hemisphere, frontal, temporal, occipital, and lateral lobes (based on FreeSurfer definition). The lobes’ area measurements were corrected by their integrated Gaussian Curvature, removing the partition size effect and enabling a direct comparison between lobes and hemisphere cortical folding^20^.

Images’ quality regarding head motion was verified during the acquisition. In cases that were identified head motion during acquisition, the team repeated the image series. Further, a senior radiologist confirmed the image quality qualitatively, approving the inclusion of the subject. Images that were not possible to process with FreeSurfer (due to head motion or other acquisition artifacts) were excluded. Due to processing errors, which would need manual intervention to be overcome, eleven subjects were excluded during the FreeSurfer processing or data extraction steps. During the visual inspection of the surfaces, images were classified with a scale ranging from 0 to 2. Images classified with 0 were rejected, and those classified as 1 and 2 can be included in the study^54^. Two subjects were classified with 0 in the MCI diagnostic group and removed from the study. One subject’s segmentation was classified as 2. Most subjects presented small portions of nonbrain regions (e.g., dura mater) included in the brain mask (72%). The final number of subjects included in this report is 121 (77 Healthy Controls, 31 with Mild Cognitive Impairment, and 13 with Alzheimer’s Disease) (Table 3).

**Table 3:** Summary of each sociodemographic, morphological, behavioral, and biochemical variables. Mean values ± standard deviation (number of subjects) and post hoc comparison of means for the demographics within groups significant difference (control group as reference).

Variable	CTL (N=77)	MCI (N=31)	AD (N=13)
Age [years]*	66 ± 8.4	72 ± 4.8*	77 ± 6.1*
Education [years]*	15 ± 2.2	13 ± 2.4*	13 ± 3*
Female, N (%)	53 (69%)	19 (61%)	8 (62%)
Morphometrical			
Cortical Thickness [mm]*	2.5 ± 0.099	2.5 ± 0.088*	2.4 ± 0.079*
Total Area [mm]	98000 ± 7800	97000 ± 8200	95000 ± 9300
Exposed Area [mm2]	37000 ± 2400	37000 ± 2600	37000 ± 3000
k*	0.30 ± 0.0095	0.29 ± 0.0094*	0.28 ± 0.01*
K ($log_{10}{k}$)*	− 0.52 ± 0.014	− 0.53 ± 0.014*	− 0.55 ± 0.015*
Behavioral			
Cognitive Index*	0.21 ± 0.64	− 1.5 ± 1.3*	− 3.4 ± 1.5*
RAVLT A7/A5*	0.82 ± 0.18	0.53 ± 0.31*	0.24 ± 0.31*
TMT B-A*	59 ± 48	130 ± 110*	230 ± 130*
Digit Span Backward*	5.8 ± 1.7	4.6 ± 1.6*	3.8 ± 1.4*
Biochemical (CSF)			
Lipoxin [pg/mL]*	130 ± 62 (28)	120 ± 51 (11)	79 ± 74 (6)*
A$\beta$1-40 [pg/mL]	4200 ± 1900 (29)	5000 ± 2600 (11)	5700 ± 1700 (6)
A$\beta$1-42 [pg/mL]*	530 ± 240 (29)	450 ± 320 (11)	280 ± 60 (6)*
t-Tau [pg/mL]*	350 ± 190 (29)	470 ± 200 (11)	630 ± 280 (6)*

*P < 0.05.

A team of physicians, psychologists, and speech therapists handled the clinical, behavioral, and biochemical assessment described and discussed previously in works by Coutinho et al.^55^ and Drummond et al.^49^. The tests included Digit Span Backwards (working memory), Rey’s Auditory Verbal Learning Test (RAVLT) A7 and A5 (memory), and Trail Making Test (TMT) (cognitive flexibility).

The RAVLT consists of five oral presentations of a word list that are repeated by the patient after each presentation (A1 to A5). After a second list is presented (as a distractor), the patient is asked to free recall (A6) the first list, which was presented and repeated five times (immediate recall). Then, after a fixed interval, the patient is again asked to repeat that first list (A7) (delayed recall). We have calculated the difference between the delayed free recall of the list (A7) and the last repetition of the list after being presented by the examiner (A5). This estimate portrays verbal memory^56^.

The Cognitive Index is calculated as a global cognitive function (composed of TMT and RAVLT) weighted for age intervals of 10 years. It is estimated as the mean value of A5, A7, TMT A, and TMT B z-scores from normative values of age and years of schooling, adapting the Cognitive Index applied in previous publications as Verburgt et al.^57^. Because both working memory and cognitive flexibility are executive functions, Digit Span Backwards was not part of the Cognitive Index to avoid over-representing executive functions in global cognition.

For a subset of our sample (28 CTL, 13 Mild Cognitive Impairment, and 6 Alzheimer’s Disease for Lipoxin, 29 CTL, 13 Mild Cognitive Impairment, and 6 Alzheimer’s Disease for A$\beta$1-42, A$\beta$1-40, and t-Tau), we included the following biochemical biomarkers from the Cerebrospinal Fluid (CSF): Lipoxin, A$\beta$1-42, A$\beta$1-40, and t-Tau. CSF biomarkers were measured from CSF samples (15 ml) extracted from lumbar punctures and using Euroimmun enzyme immunoassays with single antigen (ELISA) kits^49^.

Due to the limitation of the size sample for this study, we ran the same diagnostic discrimination analysis and optimal cut-point with a subsample of the Alzheimer’s Disease Neuroimaging Initiative (ADNI)^58^ database (adni.loni.usc.edu) included in Wang et al.^19^ for the hemispheres. The subsample included the first MRI session from subjects of ADNI 1, ADNI GO, and ADNI 2 cohorts with T1w image acquired in 3 T equipment as selected for Wang and colleagues’ study. ADNI provided images preprocessed with FreeSurfer version 5.3. The participant’s description is included in Supplementary Note 3, and the acquisition methods are in https://adni.loni.usc.edu/methods/. The ADNI was launched in 2003 as a public-private partnership, led by Principal Investigator Michael W. Weiner, MD. The primary goal of ADNI has been to test whether serial magnetic resonance imaging (MRI), positron emission tomography (PET), other biological markers, and clinical and neuropsychological assessment can be combined to measure the progression of mild cognitive impairment (MCI) and early Alzheimer’s Disease (AD).

### Data statistical analysis

To evaluate whether the dataset conforms to the universal cortical folding law (Eq. 4) fitting to the dataset included in this study, a linear regression of the logarithmic form of the expression was applied, and the R^2^ and the model slope (the self-similarity index $\alpha$) were assessed. The dataset’s slope was compared with a two-tailed Student’s t-test against the expected value of 1.25 to assess how closely the cortical folding in the dataset conforms to theoretical universal scaling law^18^.

4 $$\begin{aligned} {T^{\frac{1}{2}}A_T~=~kA_E^{\alpha }} \end{aligned}$$

We evaluated the correlation of Cortical Folding variables ($\alpha$ and $K = \log _{10}{k}$) with age using Pearson’s correlation coefficient r and Cohen’s d for the Effect Size of correlation.

Diagnostic discrimination was assessed in two aspects: the difference of means of K and Cortical Thickness, in its logarithmic form $log_{10}{T}$, between groups, and an optimal cut-point analysis for those variables. Multiple comparisons of means were made with ANOVA and post hoc pairwise evaluations with Tukey multiple comparisons of means, which presents the *P* value corrected for multiple comparisons (Bonferroni). Cut-points were determined by selecting the point with the maximum sum of sensitivity and specificity with bootstrapping to estimate cut-point variability (bootstrap number = 1000). We further compared the Area Under the Curve (AUC) of the Receiver Operating Characteristic (ROC) curve generated for each cut-point analysis, Accuracy, Sensibility, and Specificity to verify differences between morphological variables’ performance in discrimination.

Pearson’s correlation r and Cohen’s d Effect Size were calculated for correlations between morphological variables K and Cortical Thickness within Behavioral and CSF assessments. The correlation’s *P* value was corrected for multiple comparisons (Bonferroni) within the clinical assessment and morphological parameters.

All statistics were analyzed with R v4.3.0 and RStudio v2023.06.0. The statistical significance threshold was $\alpha$ = 0.05, and a 95% Confidence Interval is included for effect sizes and when necessary.

### Supplementary Information

Supplementary Information.

## Data Availability

The AOMIC and AHEAD datasets generated and/or analysed during the current study are available in the Zenodo repository, https://doi.org/10.5281/zenodo.5750619. The Wang, 2016 datasets generated and/or analysed during the current study are available in the Zenodo repository, https://zenodo.org/record/61348. The IDOR datasets generated and/or analysed during the current study are not publicly available due to local Ethics Committee approval restrictions but are available from the corresponding author on reasonable request. The R code generated and/or analysed during the current study is available in the GitHub repository, https://github.com/metaBIOlab/CorticalFolding_AD_Aging.
