# Supplementary material for: Cortical folding correlates to aging and Alzheimer’s Disease’s cognitive and CSF biomarkers
Source: Sci Rep. 2024 Feb 8;14:3222. doi: 10.1038/s41598-023-50780-3 (PMC10853184; doi:10.1038/s41598-023-50780-3)
Supplement: Supplementary file 1 — Supplementary Information. [file 41598_2023_50780_MOESM1_ESM.docx]

Supplementary Information for Cortical unfolding correlates to Aging and Alzheimer's Disease biomarkers

Fernanda Hansen P. de Moraes,^1,2^ Felipe Sudo, Marina Carneiro Monteiro, Bruno R. P. de Melo, Paulo Mattos, Bruno Mota^2,†^ and Fernanda Tovar-Moll^1,†^

^†^These authors contributed equally to this work.

Author affiliations:

1 Brain Connectivity Unit, Instituto D'Or de Pesquisa e Ensino (IDOR), Rio de Janeiro, CEP, Brazil

2 Instituto de Física, Universidade Federal do Rio de Janeiro (UFRJ), Rio de Janeiro, CEP, Brazil

3 Memory Clinic, Instituto D'Or de Pesquisa e Ensino (IDOR), Rio de Janeiro, CEP, Brazil

Correspondence to: Fernanda Tovar-Moll

E-mail fernanda.tovarmoll@idor.org

# Extended results, tables, and Figs


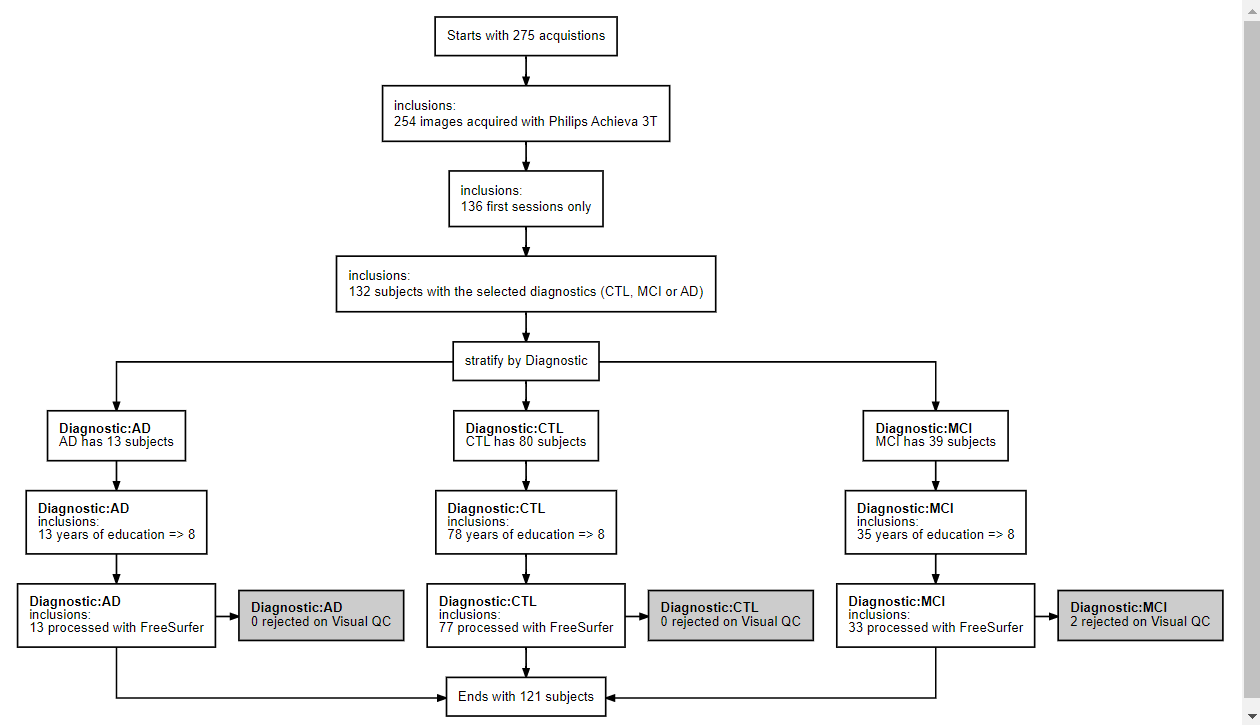


**Fig S1 Flowchart of subjects’ inclusion and exclusion criteria.**


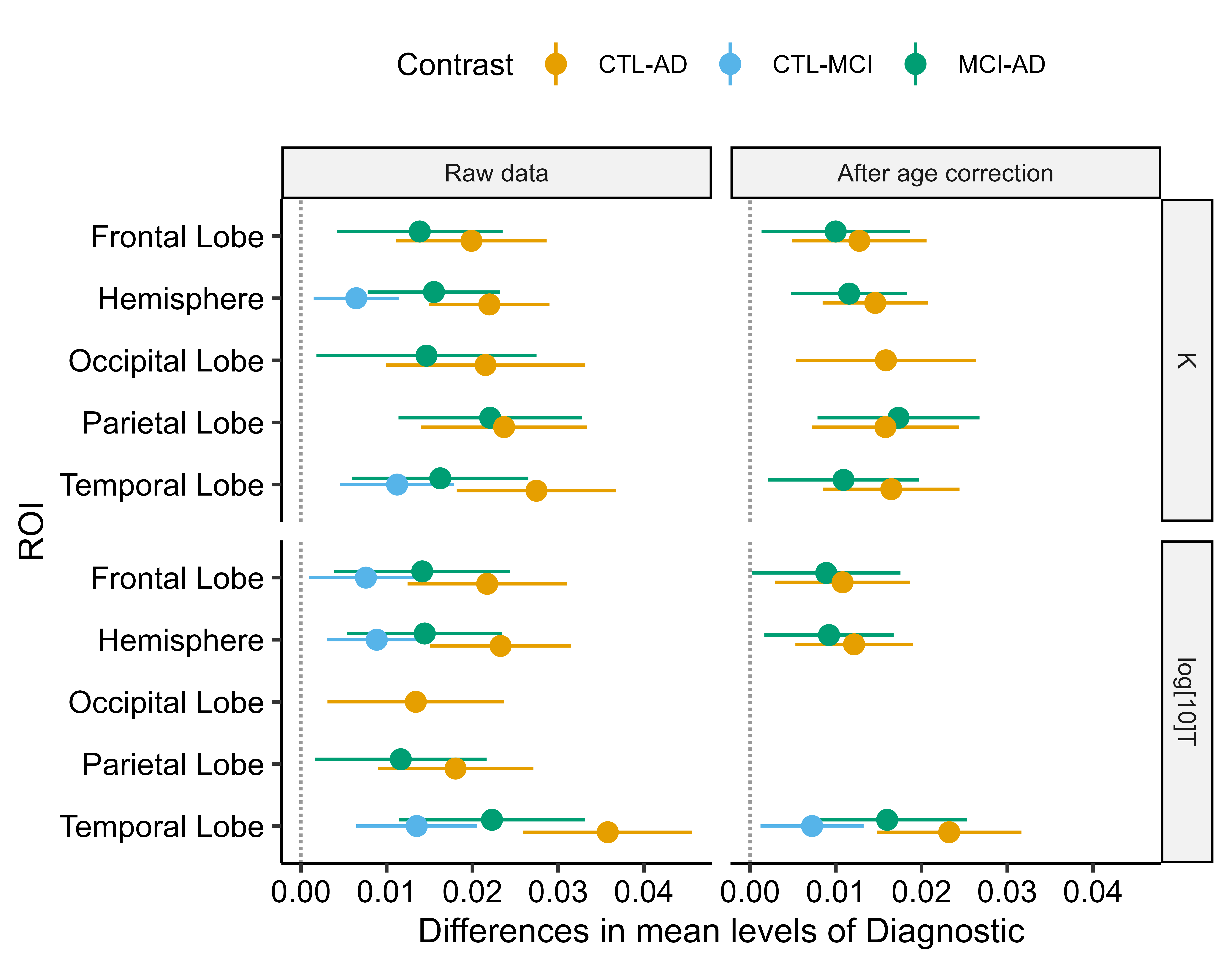


**Fig S2 Statistically significant (p < 0.05) differences in means within Diagnostic for K and log(T).** Comparisons with ("After age correction") and without ("Raw data") age correction for the hemisphere and the four lobes. Multiple corrections were applied within each morphological feature and ROI. Bars represent a 95% Confidence Interval.





**Fig S3 Difference of means in pairwise comparison for AD-CTL, AD-MCI, and MCI-CTL grouped by age in decades in each ROI.** (**A**) K and (**B**) K after age correction. Bars represent a 95% confidence interval. There is no statistical power to infer that the difference between diagnostics is more significant in younger adults, probably influenced by the small number of observations in each data point.


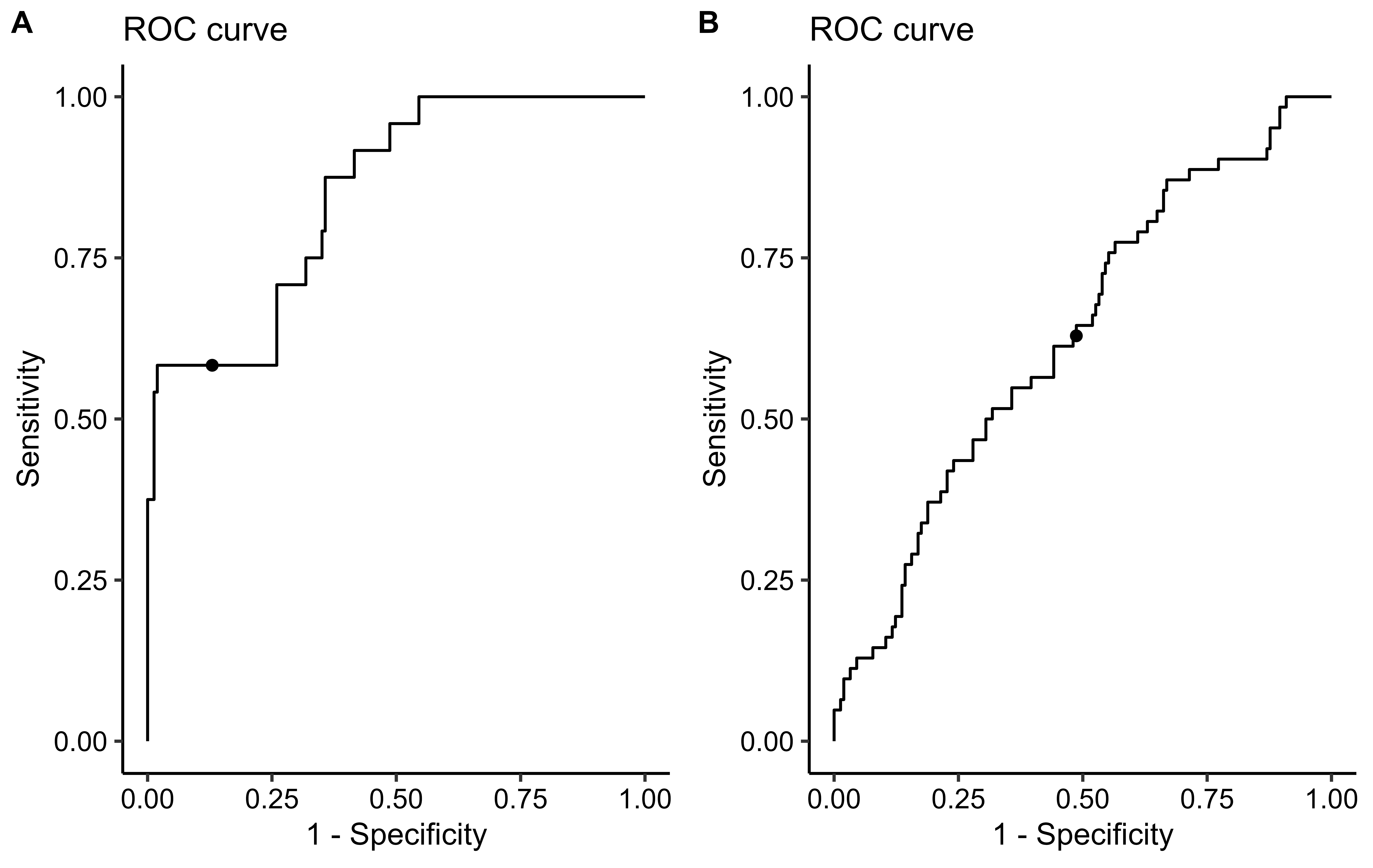


**Fig S4 ROC curve from the optimal cut-off analysis of K (hemisphere as ROI).** (**A**) Discriminating CTL and AD subjects, and (**B**) discriminating CTL and MCI subjects.

**Table S1 Optimal cut point (maximum sensitivity and specificity) for K at each ROI (hemispheres and lobes).** Cut point AUC, Accuracy, sensibility, and specificity for discriminating pairwise diagnostic groups.

| **ROI** | **CTL-AD** | | | | | **CTL-MCI** | | | | |
| --- | --- | --- | --- | --- | --- | --- | --- | --- | --- | --- |
|  | **Cut point** | **AUC** | **ACC** | **SENS** | **SPEC** | **Cut point** | **AUC** | **ACC** | **SENS** | **SPEC** |
| H | -0.54 | 0.84 | 0.82 | 0.58 | 0.86 | -0.53 | 0.63 | 0.60 | 0.56 | 0.56 |
| F | -0.55 | 0.75 | 0.69 | 0.65 | 0.69 | -0.54 | 0.60 | 0.55 | 0.67 | 0.50 |
| O | -0.50 | 0.69 | 0.79 | 0.58 | 0.58 | -0.48 | 0.56 | 0.49 | 0.72 | 0.40 |
| P | -0.52 | 0.82 | 0.75 | 0.77 | 0.75 | -0.50 | 0.51 | 0.47 | 0.57 | 0.43 |
| T | -0.51 | 0.81 | 0.69 | 0.73 | 0.68 | -0.51 | 0.67 | 0.59 | 0.66 | 0.56 |
| AUC – Area Under the Curve, ACC - Accuracy, SENS – Sensibility, and SPEC - Specificity.  ROI codes: H - Hemisphere, F - Frontal, O - Occipital, P - Parietal, and T - Temporal lobes. | | | | | | | | | | |


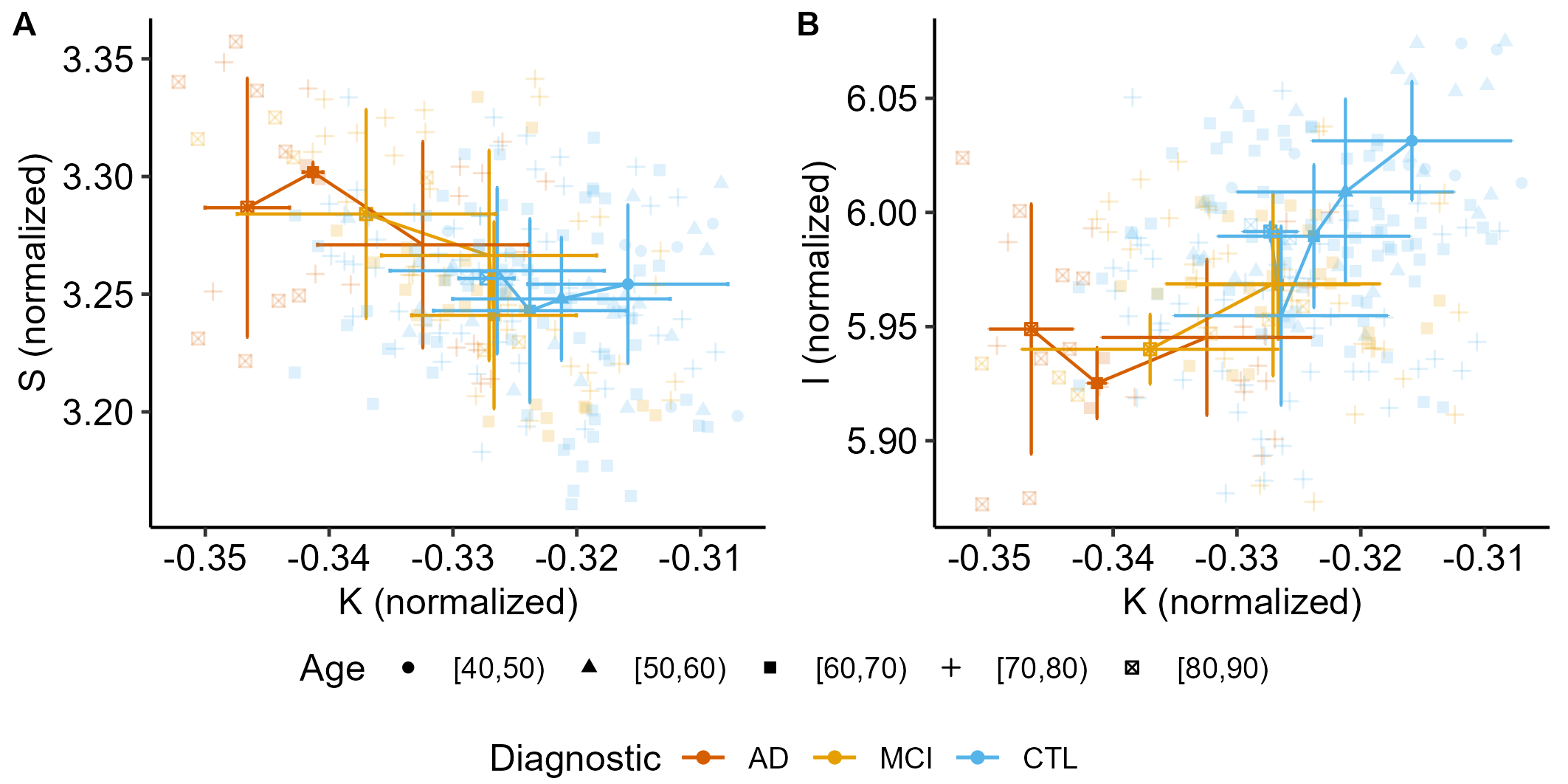


**Fig S5 Morphological trajectory traced across the normalized independent components K, S, and I.** We normalized the variable to the unity vectors providing a comparable scale for the differences in both axes. Bars represent the standard deviation of means. Diagnostics groups were divided into subgroups of ten years intervals. **(A)** K and S trajectory and **(B)** K and I trajectory.

**Table S2 Extended table with Pearson's r correlation results and Cohen’s d effect size for behavioral assessments and morphological parameters, K and Cortical Thickness, with and without age correction.** The P-value was corrected for multiple comparisons (Bonferroni) within Clinical Assessment and morphological measurement.

| **Clinical Assessment** | **Morphological measurement** | **K [r; d]** | | **Log_10_(T) [r; d]** | |
| --- | --- | --- | --- | --- | --- |
|  | **Age correction/ROI** | **no** | **yes** | **no** | **yes** |
| Cognitive Index | H | *0.41*; 0.9* | *0.31*; 0.65* | *0.40*; 0.87* | *0.26*; 0.54* |
| RAVLT A7/A5 | H | *0.36*; 0.77* | *0.27*; 0.56* | *0.39*; 0.85* | *0.26*; 0.54* |
|  | T | *0.31*; 0.65* | *0.23*; 0.47* | *0.44*; 0.98* | *0.34*; 0.72* |
| TMT B-A | H | *-0.30*; -0.63* | *-0.20*; -0.41* | *-0.22*; -0.45* | -0.069; -0.14 |
|  | F | *-0.21*; -0.43* | -0.11; -0.22 | *-0.21*; -0.43* | -0.08; -0.16 |
| Digit Span Backwards | H | *0.25*; 0.52* | *0.19*; 0.39* | *0.20*; 0.41* | 0.11; 0.22 |
|  | F | *0.21*; 0.43* | *0.17*; 0.35* | *0.21*; 0.43* | 0.074; 0.15 |
| Aβ1-40 | H | -0.073; -0.15 | -0.018; -0.04 | *-0.22*; -0.45* | -0.16; -0.32 |
| Aβ1-42 | H | *0.26*; 0.54* | 0.26*; 0.54 | 0.086; 0.17 | -0.001; 0.00 |
| t-Tau | H | *-0.26*; -0.54* | -0.18; -0.37 | *-0.41*; -0.9* | *-0.28*; -0.58* |
| Aβ1-42/Aβ1-40 | H | 0.18; 0.37 | 0.16 0.32 | 0.20; 0.41 | 0.14; 0.28 |
| t-Tau/Aβ1-42 | H | *-0.32*; -0.68* | -0.24; -0.49 | *-0.34*; -0.72* | -0.17; -0.35 |
| t-Tau/(Aβ1-42/Aβ1-40) | H | *-0.28*; -0.58* | -0.2; -0.41 | *-0.34*; -0.72* | *-0.23*; -0.47* |
| Lipoxin | H | 0.11; 0.22 | 0.13; 0.26 | -0.052; -0.10 | -0.046; -0.09 |
| **P < 0.05.*  ROI codes: H - Hemisphere, F - Frontal Lobe, and T – Temporal Lobe. | | | | | |

# Quality Control of generated surfaces

One usual quantitative quality control index for Surface-based morphometry is the Euler number, which represents the number of handles, holes, and defects in a surface. In the FreeSurfer pipeline is also possible to estimate the Euler number before the automatic surface correction. In a recent paper by Rutherford and colleagues, they suggest using the Euler number to indicate which images should be visually inspected in the context of big datasets that are humanly impossible to verify all images (Rutherford et al., 2022).

In our sample, we estimated the Euler number as defined by the FreeSurfer team, Euler number = 2- 2*n, where n is the number of holes. As the FreeSurfer team indicates, the number of holes is extracted by the *asegstats* file of each subject. A result that differs from 2 indicates that the surface is not topologically equivalent to a sphere^[[1]](#footnote-2)^. For the IDOR sample, all subjects presented an Euler number equal to 2 in the fixed/final surfaces, meaning no topological errors. For the surfaces before correction, the mean Euler number between hemispheres was negative, indicating the presence of holes or handles, with a mean value of -51.8 ± 18.8 (AD: -55.4 ± 18.7; MCI: -53.9 = 22.1; CTL: -50.2 ± 17). There is no difference between diagnostic group means (ANOVA F = 0.78, DF = 2, P > 046, P > 0.05 for all pairwise comparisons), and one MCI subject was noted as an outlier in its group. Thus, we included a visual inspection step of each surface and subject as a quality control measurement using the suggested rate scale from Rosen and colleagues (Rosen et al., 2018), ranging from 0 to 2. Surfaces classified as 1 and 2 can be included in the study, while surfaces with 0 cannot. 2 MCI subjects (one of them indicated as an outlier in the Euler number inspection) were classified in the rejection category, 97.5% of the surfaces were classified with 1, and 0.83% with 2. The main errors in surface correction were due to the inclusion of a small portion of dura matter (72%). Since these errors were equally distributed across diagnostics, there should be no bias in the analysis presented in this manuscript.

Nevertheless, we acknowledge the fact that there is a lack of widespread quantitative quality control measurements for surface-based MRI morphology, which must include not only the number of handles and holes in a surface but also if the whole brain was included, and a measurement to verify the correctness of the white/grey matter and grey matter/CSF boundaries.

# Slope interpretation

One unexpected aspect of the reported data in the manuscript is that the data presents a smaller cortical folding model slope (Fig S6) than the previously published results (Wang et al., 2016). Considering that multiple acquisition and processing sites increase the variability in brain morphology studies (Chepkoech et al., 2016; Dickerson et al., 2008; Fortin et al., 2018; Gronenschild et al., 2012; Heinen et al., 2016), the slope analysis should be handled by reducing the possible methodological confounding variables.


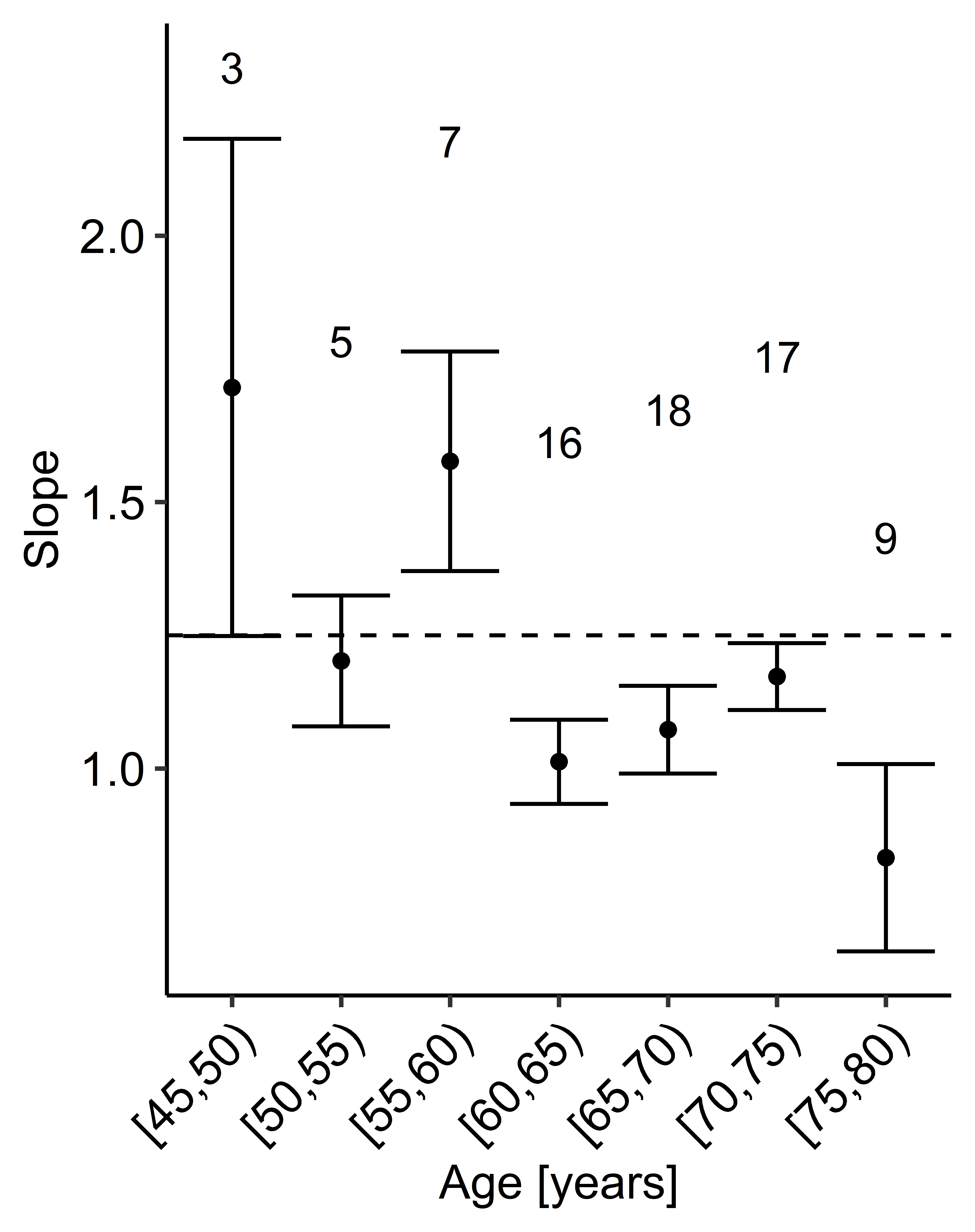


**Fig S6 Age effect in cortical gyrification.** Cortical folding model slope α within five years intervals for Healthy Cognitive Unimpaired Controls. Above each point, we display the number of subjects in each regression. Bars represent the standard deviation for the respective age interval regression. Points with one subject were excluded due to the lack of statistical significance.

We compared the data of this manuscript with the Amsterdam Open MRI Collection (AOMIC) PIOP01 dataset (Snoek et al., 2021), at which T1-weighted MRI were acquired with the same equipment, Philips Achieva 3 T. Moreover, we cross-validated the results with the Amsterdam Ultra-High Field Adult Lifespan database (AHEAD), a 7 T available set of MR images (Alkemade et al., 2020).

AOMIC PIOP01 and AHEAD were processed with the FreeSurfer v6.0 (Fischl, 2012) standard pipeline, the localGI pipeline, and the Cortical Folding Analysis Tool(Wang et al., 2019). The AOMIC PIOP01 dataset contains 216 healthy younger subjects with an average age of 22 ± 1.8 years old, close to 25 years old, the age used to correct our data. Seven subjects were excluded for missing age information, and one was due to processing and extraction errors. AHEAD dataset contains 105 healthy subjects from 18 to 80 years old. From those, four subjects were excluded due to errors in FreeSurfer processing or needed of manual surface correction. Processed data from both datasets are available (de Moraes, 2021) (<https://doi.org/10.5281/zenodo.5750619>). We included S and I values for the datasets in the work by Wang et al. (Wang et al., 2016) (Table S3).

**Table S3 Description of the datasets included in this report, summarizing each morphological, cognitive, and biochemical test.** Values are mean values ± standard deviation.

| **Sample** | **N (F)** | **Diagnostic** | **Age (range) [years]** | **MRI Equipment** | **FreeSurfer version** |
| --- | --- | --- | --- | --- | --- |
| IDOR | 77 (53) | CTL | 66 ± 8.4 (43-80) | Philips Achieva 3T | 6.0 |
|  | 31 (19) | MCI | 72 ± 4.8 (62-82) |  |  |
|  | 13 (8) | AD | 77 ± 6.1 (63-85) |  |  |
| ADNI | 215 (115) | CTL | 74 ± 6.9 (56-95) | 3T Different across sites | 5.3 |
|  | 196 (90) | AD | 74.9 ± 8.2 (56-91) |  | 5.3 modified version |
| AOMIC PIOP01 | 208 (84) | CTL | 22 ± 1.8 (18-26) | Philips Achieva 3T | 6.0 |
| AHEAD | 102 (58) | CTL | 43 ± 19 (18-80) | Philips Achieva 7T | 6.0 |
| HCP500r | 508 (301) | CTL | 29 ± 3.4 (22-36) | Siemens Skyra (modified) 3T | 5.3 modified version |
| NKI | 168 (68) | CTL | 34 ± 19 (4-85) | Siemens Magnetom 3T | 5.0 |
| OASIS | 312 (196) | CTL | 45.0 ± 24 (18-94) | Siemens Vision Scanner 1.5T | Dev 20061005 |
| **P < 0.05.*  ROI codes: H - Hemisphere, F - Frontal Lobe, and T – Temporal Lobe. | | | | | |

The IDOR slope is smaller than HCP500r, AHEAD, AOMIC, NKI, and OASIS and comparable to ADNI-Control and ADNI-AD, both samples with exclusively elder subjects (Fig S7). To confirm the hypothesis of slope dependency on age without the confounding methodological variables, we verified the slope behavior in AOMIC and IDOR-Control samples through Age (Fig S8-A). There is a tendency for a reduced slope with the increase of age, which is similar to the negative gradient found in cortical thickness and K (Figs S8-B and C). These results are validated when compared to the AHEAD subjects (Fig. S8). After a certain age, between 40 to 60 years, the slope escapes the model’s expectations, which could be explained by the nonhomogeneity in aging effects on brain structure. Aging occurs with different onsets, locations, and scales (from a single cerebral gyrus to a whole lobe).

Future works with data from a unique site and protocol could evaluate socioeconomic status's effect and prospect the slope's biological meaning.


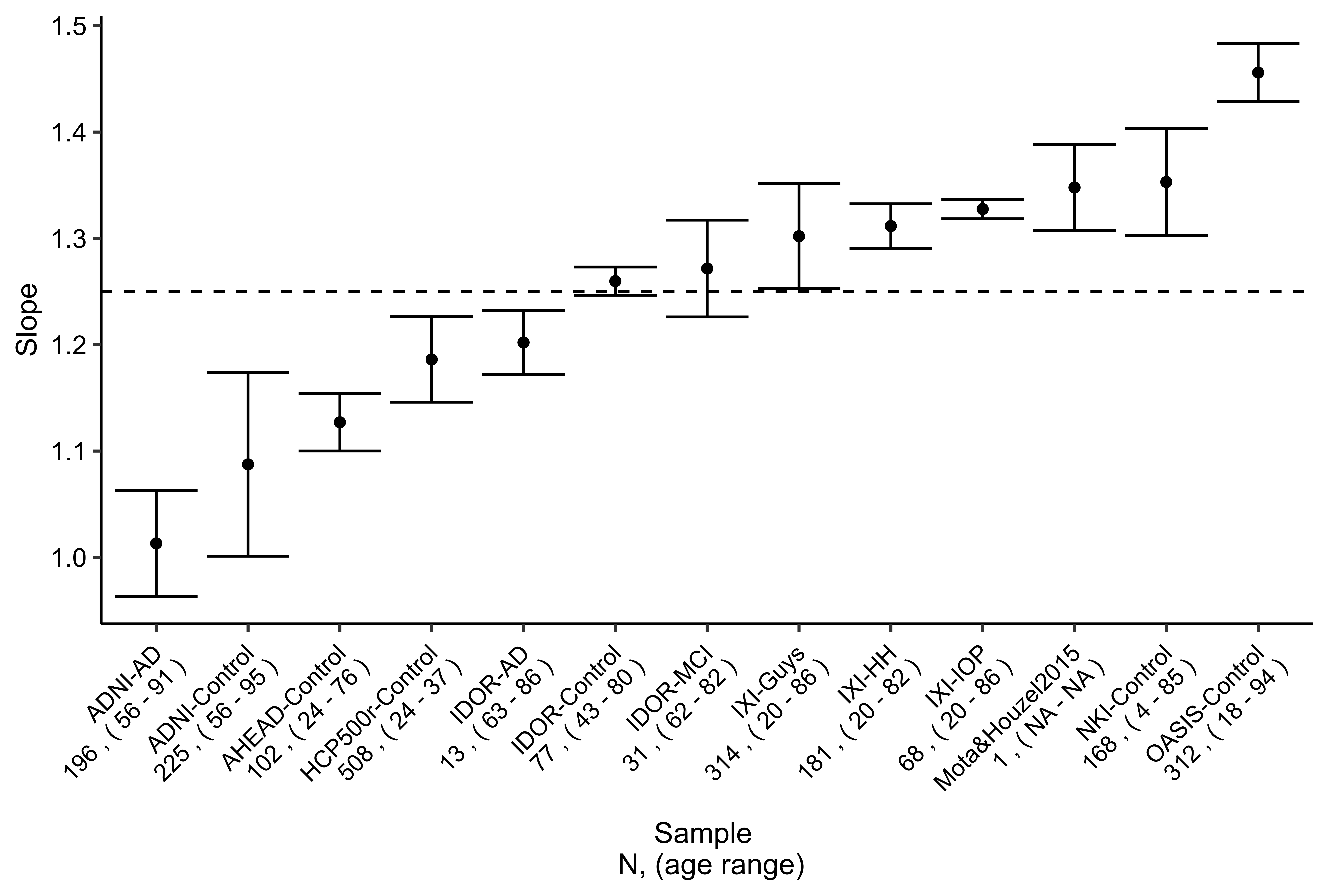


**Fig S7 Slope αfor each sample.** The traced line is for 1.25, the theoretical value of the slope α. Bars represent the standard deviation. Bellow the sample is presented the number of subjects included and the age range.


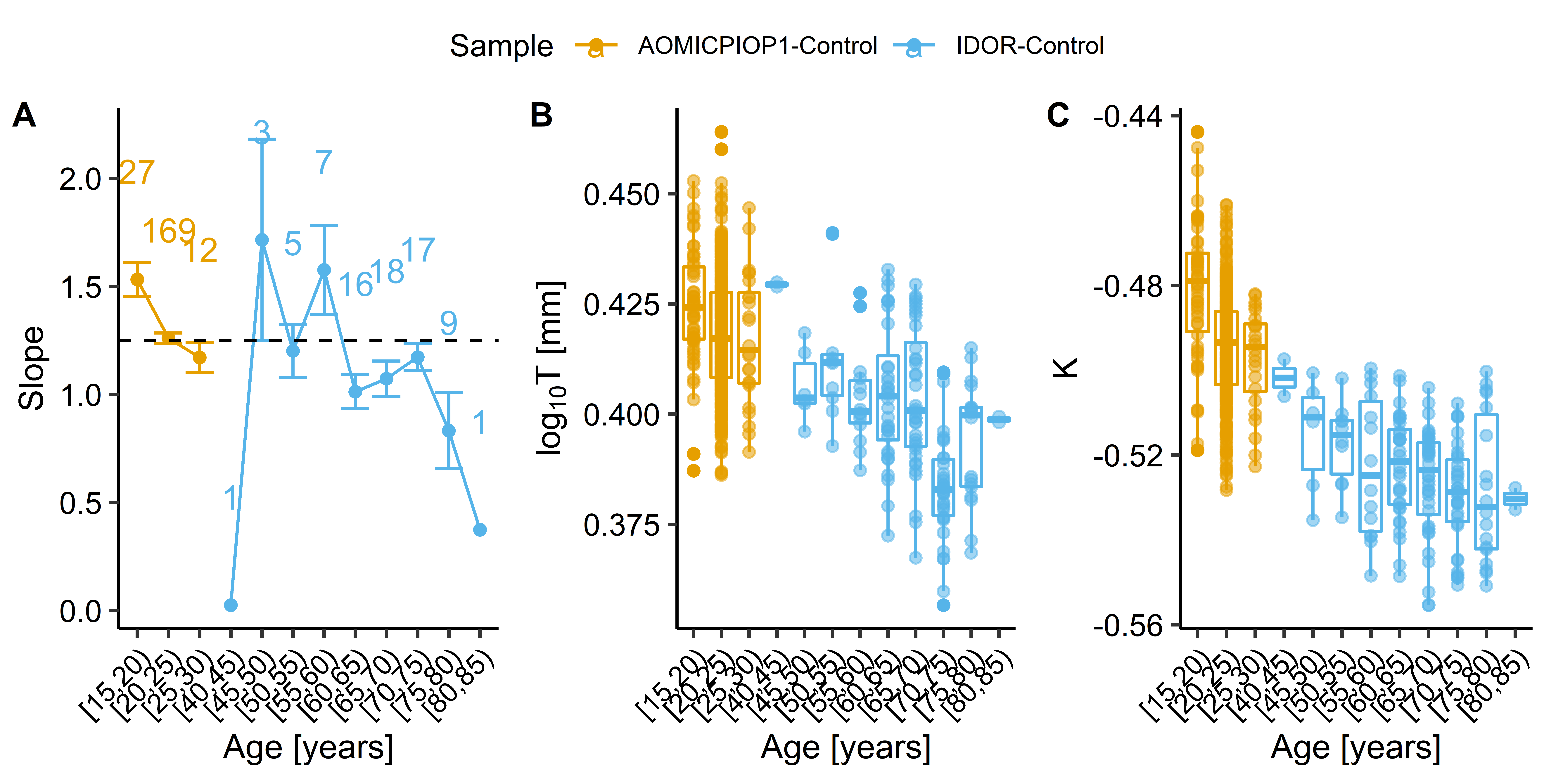


**Fig S8 Plots comparing morphological variables’ behavior with aging.** Yellow is for AOMICPIOP01-Control, and blue is for IDOR-Control. The x-axis is modified to hide the age interval from 30 to 40 years with no subjects, two data points with only one subject, 40-45 and 80-85. The total number of subjects included is 284. **(A)** The slope for each Age interval group and each sample. The number on top of the point indicates the number of subjects included in the linear regression, and each subject contributes with two data points, one for each hemisphere. The traced line represents the slope theoretical value of 1.25. Bars represent the standard deviation. **(B)** Log_10_(T) distribution for each Age interval group and each sample. Bars represent a 95% confidence interval. **(C)** K distribution for each age interval group and each sample. Bars represent a 95% confidence interval.


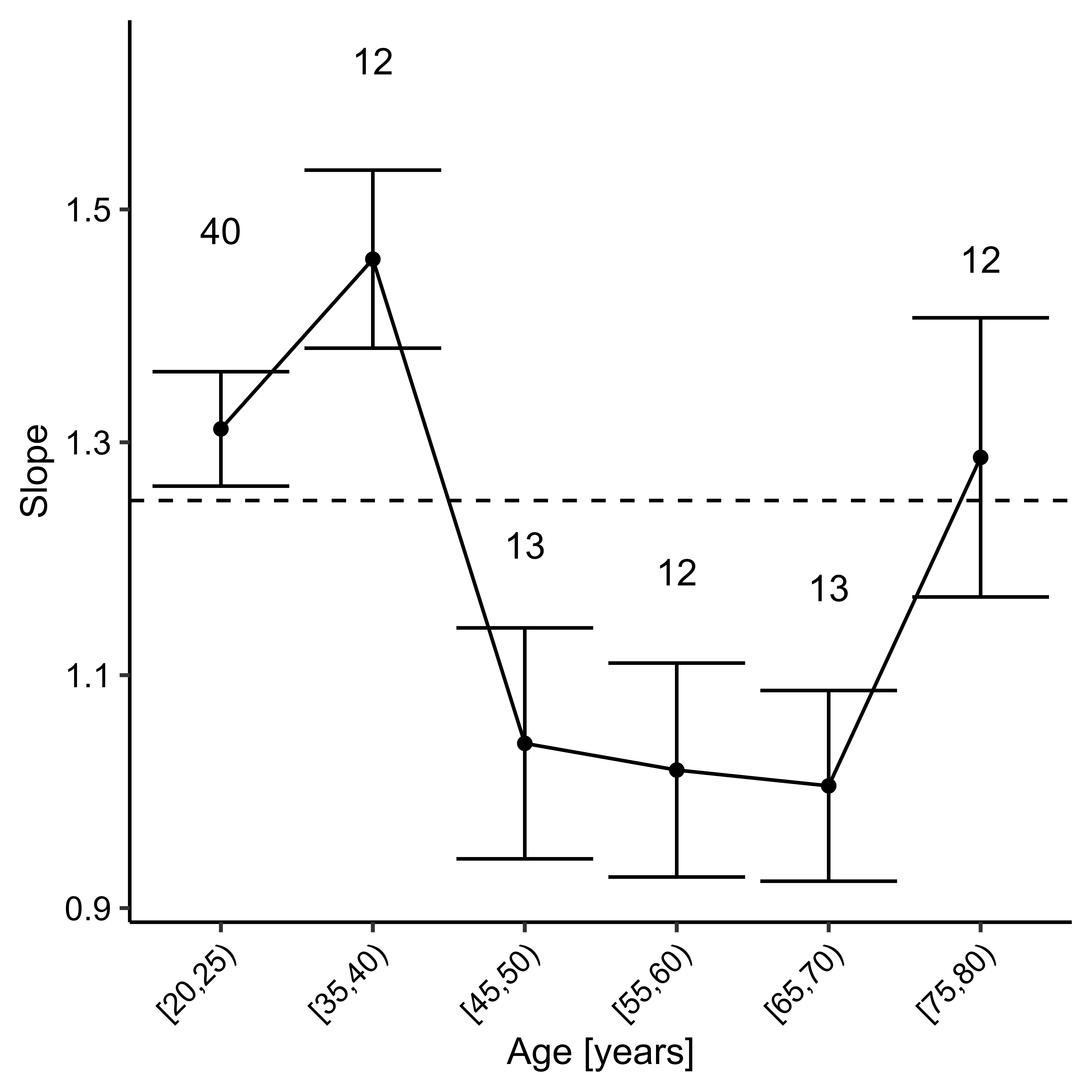


**Fig S9 Slope for each age interval group in AHEAD-Control.** The number on top of the point indicates the number of subjects included in the linear regression, and each subject contributes with two data points, one for each hemisphere. The traced line is for 1.25, the theoretical value of the slope α. Bars represent the standard deviation.

# Gender and Education as potential confounders

Due to the complexity of Alzheimer’s Disease (Frozza et al., 2018), which is known to be closely related to aging, metabolic disorders such as impaired insulin signaling (De Felice et al., 2022), chronic inflammation process resolution (Gonçalves et al., 2022; Pamplona et al., 2022; Whittington et al., 2017), and cognitive reserve/protection (Kim et al., 2015; Liu et al., 2012; Stern, 2012). Acknowledging the inherent complexity of the studied pathology, we intend to show that Sex/Gender and Years of Education are not potential confounders in the found results.

In both situations, we verified the influence of a covariate in a linear regression with K (with and without age correction), and Diagnostic, the residuals from the generated model and the variance accounted for the covariate. Both Gender and Years of Education imply in significant but small influence on K and K (age-corrected); results are summarized in Table S4, and residuals in Fig S10.

**Table S4 Summary of confounders’ influence in K and K (age-corrected) including Coefficient estimates, standard errors, t value, P value, and the variance explained by the confounders (R²) extracted from the linear models.**

| **Variable** | **Estimate** | **Std. Error** | **t value** | **Pr(>\|t\|)** | **Variance explained by (adjusted R²)** |
| --- | --- | --- | --- | --- | --- |
| **Gender** |  |  |  |  |  |
| K | -0.0049 | 0.0019 | -2.60 | *0.0099** | 0.026 |
| K (age-corrected) | -0.0052 | 0.0016 | -3.175 | *0.0017** | 0.039 |
| **Education** |  |  |  |  |  |
| K | -0.00039 | 0.00039 | -1.008 | 0.315 | 0.007 |
| K (age-corrected) | -0.00068 | 0.00034 | -2.030 | *0.0435** | -0.0042 |
| **P < 0.05.* | | | | | |

The results show that despite the significant influence, the variance of K and K (Age corrected) explained by Gender is 2.6% and 3.9%. The effect is even small for Education, 0.7% and 0.42%. To reinforce the slight difference between Genders, the work of Wang and colleagues, 2016 argues that the brain of males and females folds in the same manner, assessed with the cortical folding model, besides the differences in volume and a small difference in the Gyrification Index, meaning there is just one universal rule for folding, and this rule includes both male and females.

Therefore, we did not include Gender and Education as potential confounders in the main analysis. Still, we do acknowledge there are minor effects from both in the cortical folding variable studied, K.


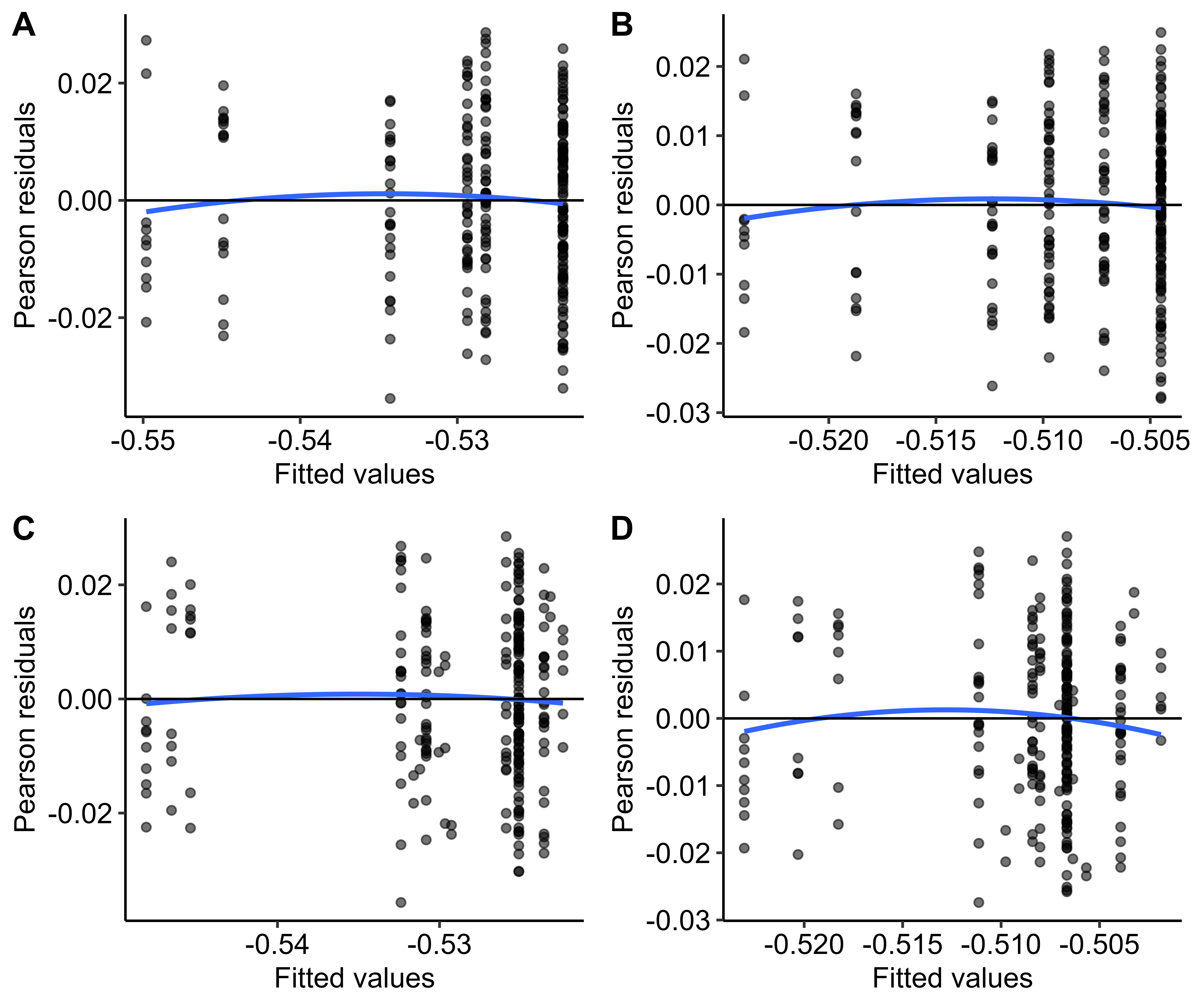


**Fig S10 Residuals plots from models testing the inference of Gender and Education on K values.** (A) Gender interaction with K; (B) Gender interaction with K (age-corrected); (C) Education interaction with K; and (D) Education interaction with K (age-corrected).

# Validation of results with ADNI subsample

We intended to provide more information due to limitations regarding the limited sample size. Thus, we included the subsample of the Alzheimer’s Disease Neuroimaging Initiative (ADNI)^[[2]](#footnote-3)^ database ([adni.loni.usc.edu](file:///D:\idor\apresentações\Artigos\Submissao%20Brain%20-%20Cortical%20Folding%20AD%20and%20Aging\adni.loni.usc.edu)) included at Wang et al. (Wang et al., 2016) with 225 Healthy Controls subjects and 196 with Alzheimer’s Disease (Table S5).

**Table S5 Summary of each sociodemographic and morphological variables from ADNI.** Mean values ± standard deviation (number of subjects) and post hoc comparison of means for the demographics within groups significant difference (control group as reference).

| **Variable** | **CTL (N=225)** | **AD (N=196)** |
| --- | --- | --- |
| Age [years] | 74 ± 6.9 | 75 ± 8.2 |
| Female, N (%) | 119 (53%) | 90 (46%) |
| **Morphometrical** | | |
| *Cortical Thickness [mm]** | *2.3 ± 0.11* | *2.2 ± 0.13* |
| Total Area [mm] | 96000 ± 8500 | 95000 ± 10000 |
| Exposed Area [mm^2^] | 39000 ± 2700 | 39000 ± 3200 |
| *k** | *1.1 ± 0.026* | *1.1 ± 0.032* |
| *K (log_10_k)** | *-0.57 ± 0.018* | *-0.59 ± 0.022* |
| *S** | *9.3 ± 0.13* | *9.4 ± 0.14* |
| *I [mm^6^]** | *10 ± 0.075* | *10 ± 0.098* |
| **P < 0.05.*  Diagnostic code: CTL for Controls and AD for Alzheimer’s Disease. | | |

## Results

The data fits the model with α = 1.17 ± 0.02 [1.12; 1.21] (R² = 0.77, p < 0.001), statistically different from the theoretical value, 1.25 (Student’s t = 3.55, p = 0.0004). There is a significant indication that Healthy aging reduces brain gyrification in terms of α (Pearson’s r = -0.32, DF = 7, p = 0.0345, d = 1.54 [-1.1, 4.19]) in this ADNI sub-sample, confirming the result presented in the main text, at which there is a tendency of reduced α value with aging. Healthy aging leads to a less folded brain in terms of K (Pearson’s r = -0.39, DF = 448, p < 0.0001, d = -0.84 [-1.12, -0.55]) (Fig S11) and I (Pearson’s r = -0.26, DF = 448, p < 0.0001, d = -0.53 [-0.8, -0.26]), but not in S (Pearson’s r = 0.27, DF = 448, p < 0.0001, d = 0.55 [0.28, 0.83]).


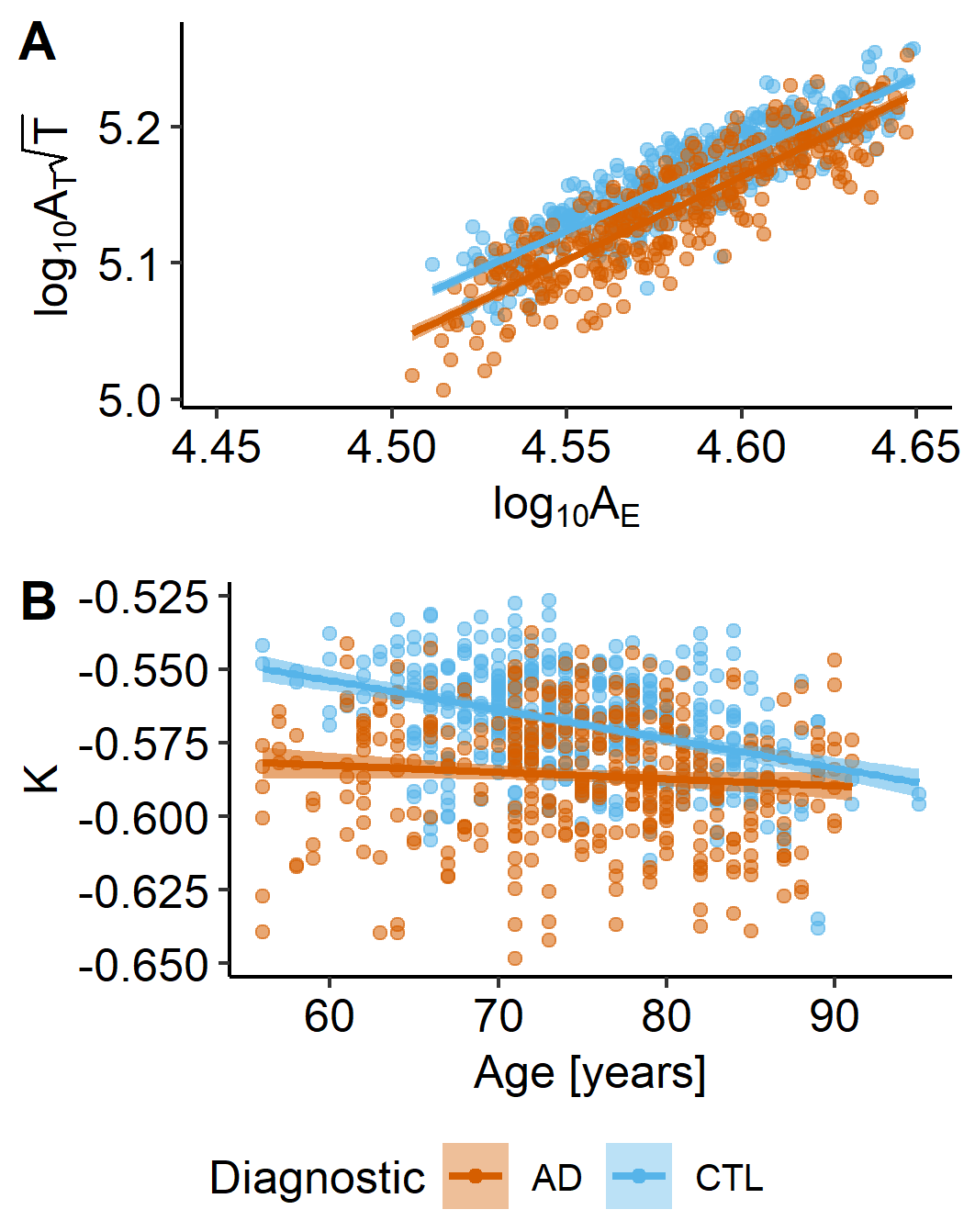


**Fig S11 Cortical folding across Diagnostics and Age. (A)** Linear fitting with a 95% Confidence Interval (CI) for the model variables in each Diagnostic group, CTL (adjusted R^2^ = 0.80, p < 0.0001) and AD (adjusted R² = 0.80, p < 0.0001). As the severity of the disease increase, the linear tendency is downshifted, with smaller linear intercepts (K). **(B)** K linear tendency across age with 95% CI for the diagnostics groups: AD (adjusted R² = .0075, p = 0.086) and CTL (adjusted R² = 0.15, p < 0.0001).

### Diagnostic discrimination

We confirm the difference in K between Healthy Controls and Alzheimer’s Disease groups (Welch t-test t = -13.1, DF = 761.46, p < 0.0001), meaning a global structural change with the pathology.

We evaluated K and Cortical Thickness (log_10_T) optimal cut points in raw data and, after age correction, to compare their discriminative power (Fig S12) as done for the IDOR sample. K optimal cut point for discriminating AD and Healthy Controls is -0.57 with 66% of accuracy (AUC = 0.74, specificity = 0.59, sensitivity = 0.75), while log_10_T presents 72% accuracy (cut point = 0.35, AUC = 0.78, specificity = 0.79, sensitivity = 0.64). Age correction here does not lead to much improvement in this analysis, in either K (cut point = -0.53, AUC = 0.72, accuracy = 0.66, specificity = 0.61, sensitivity = 0.70) or log_10_T (cut point = 0.40, AUC = 0.78, accuracy = 0.71, specificity = 0.60, sensitivity = 0.82), except for log_10_T sensitivity.


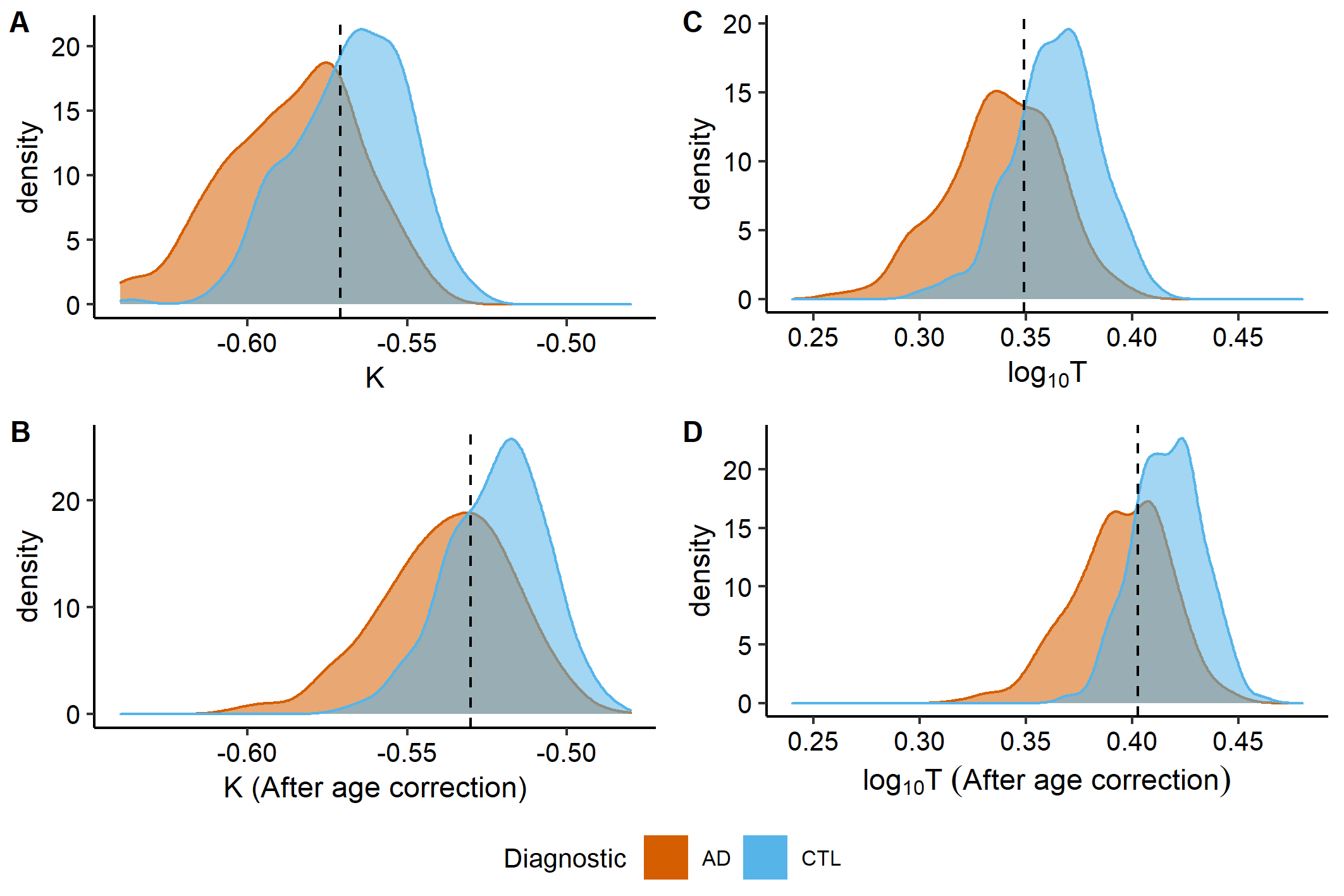


**Fig S12 Optimal cut-off (maximum sensitivity and specificity) for K and Cortical Thickness, including results with removed age effect ("age correction").** The dashed line represents the optimal cut-off to discriminate between AD and CTL. **(A)** For K, the optimal cut-off for the CTL-AD contrast is -0.57. **(B)** For K, after age correction, the optimal cut-off for CTL-AD = -0.53. **(C)** For log_10_T, the optimal cut-off for CTL-AD = 0.35 mm. **(D)** For log_10_T, after age correction, the optimal cut-off for CTL-AD = 40 mm.

### Aging and pathological morphology alterations

Extending the morphological trajectories analysis to the ADNI subsample ANOVA showed a significant effect on the group interaction of Diagnostic and Age in the tension component K (F = 74.1, DF = 3, p < 0.0001), the shape component S (F = 57.1, DF = 3, p < 0.0001), and I (F = 36.8, DF = 3, p < 0.0001), the isometric volume component.

The trajectories suggest that the AD group reaches a more aged brain stage for variables related to axonal tension and cortical shape (K and S), with the most noticeable changes in K (Table S6 and Fig S13).

**Table S6 Summary of each sociodemographic and morphological variables from ADNI. Mean values ± standard deviation (number of subjects) and post hoc comparison of means for the demographics within groups significant difference (control group as reference).**

| **Sample** | **Diagnostic** | **Age interval** | **N** | **Age** |
| --- | --- | --- | --- | --- |
| ADNI | CTL | [50,60) | 2 | 57 ± 1.2 |
|  |  | [60,70) | 51 | 66 ± 2.3 |
|  |  | [70,80) | 119 | 74 ± 2.9 |
|  |  | [80,90) | 51 | 84 ± 2.5 |
|  |  | [90,100] | 2 | 93 ± 2.3 |
|  | AD | [50,60) | 9 | 57 ± 1.2 |
|  |  | [60,70) | 36 | 65 ± 2.6 |
|  |  | [70,80) | 94 | 75 ± 2.8 |
|  |  | [80,90) | 52 | 84 ± 2.9 |
|  |  | [90,100] | 5 | 90 ± 0.42 |
| IDOR | CTL | [40,50) | 4 | 47 ± 2.5 |
|  |  | [50,60) | 12 | 55 ± 3.5 |
|  |  | [60,70) | 34 | 66 ± 2.9 |
|  |  | [70,80) | 26 | 74 ± 2.5 |
|  |  | [80,90) | 1 | 80 ± 0 |
|  | MCI | [60,70) | 11 | 68 ± 2.4 |
|  |  | [70,80) | 17 | 74 ± 2.6 |
|  |  | [80,90) | 3 | 81 ± 0.87 |
|  | AD | [60,70) | 1 | 63 ± 0 |
|  |  | [70,80) | 8 | 76 ± 4.3 |
|  |  | [80,90) | 4 | 83 ± 2.1 |
| Diagnostic code: CTL for Controls, MCI for Mild Cognitive Impairment, and AD for Alzheimer’s Disease. | | | | |


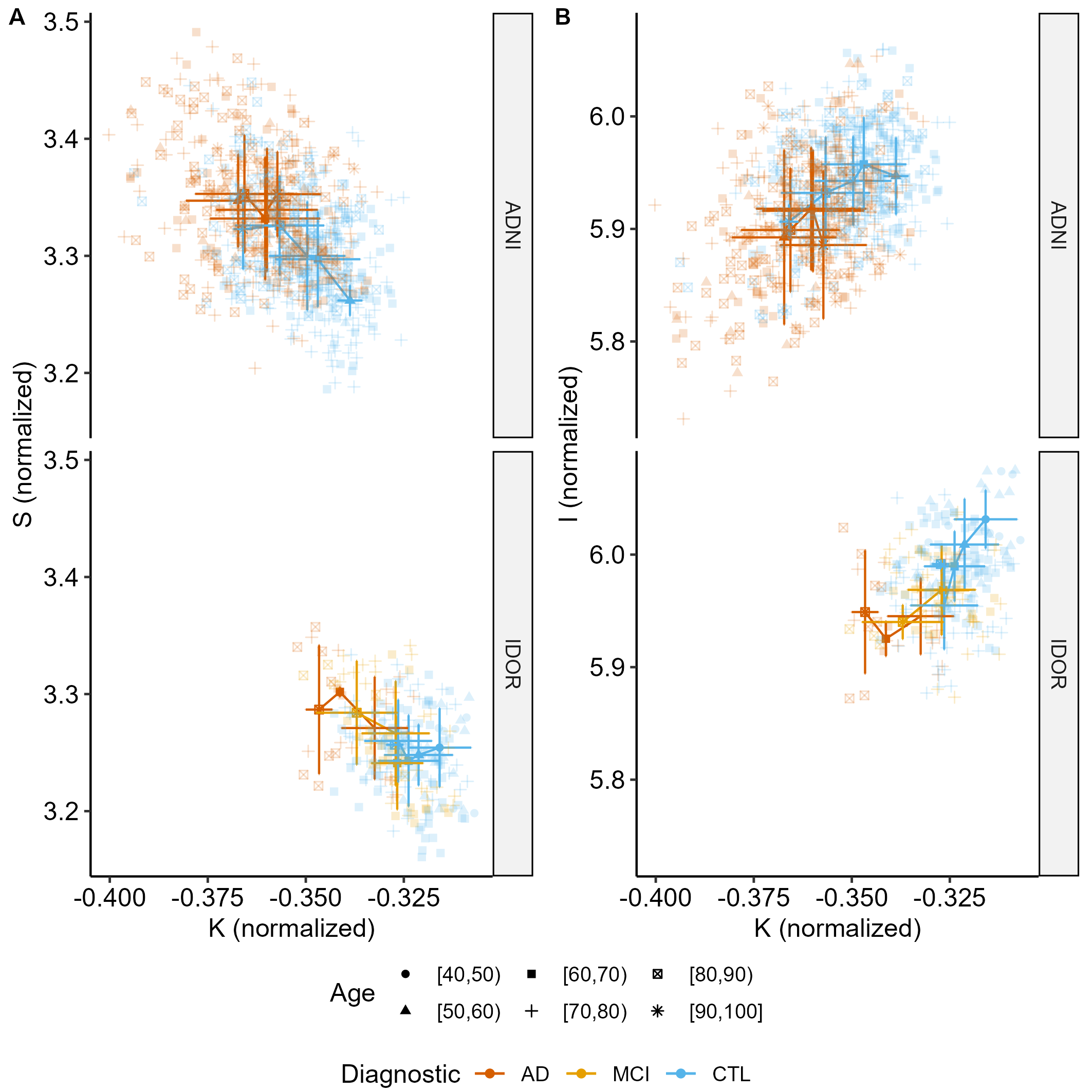


**Fig S13 Morphological trajectory traced across the normalized independent components K, S, and I.** We normalized the variable to the unity vectors providing a comparable scale for the differences in both axes. Groups were divided into subgroups. **(A)** K and S trajectory and **(B)** K and I trajectory.

# Bimodal distribution of K in Alzheimer's Disease and optimal cut-off analysis

As commented in the Manuscript Discussion, the bimodal distribution of K is very prominent for the AD subjects. However, the reduced number of subjects limits our statistical and inference power. Nevertheless, the bimodal distribution communicates that two groups are divided by the intensity of whole-brain structural injuries in this sample. Lower values of K are related to healthy aging and Alzheimer's Disease, as proved here and in a previous work by Wang et al. (Wang et al., 2016). Based on Alzheimer's Disease spatial evolution in the human brain (Frisoni et al., 2010), one could hypothesize that the worst the structural damage, the spreader the damage would be, and the lower K values would be found. From this hypothesis, we estimated the minimum value of the valley between peaks with a simple mathematical procedure (value of K were f'(K) = 0. The valley is placed at K = -0.5432, with 11 hemispheres with K > -0.5432 and 15 hemispheres with K < -0.5432, with 26 hemispheres for the 13 subjects (Fig S14).





**Fig S14 K density plots across hemispheres and lobes.** (**A**) AD is red, MCI is yellow, and CTL is blue. All AD subjects were included in the first column, with all MCI and CTL subjects. The second column display results only for AD subjects with hemispherical K < -0.54 and MCI and CTL subjects. Finally, all AD subjects with hemispherical K > -0.54 or K = -0.54 with MCI and CTL subjects.

# References

Alkemade, A., Mulder, M. J., Groot, J. M., Isaacs, B. R., van Berendonk, N., Lute, N., Isherwood, S. J., Bazin, P.-L., & Forstmann, B. U. (2020). The Amsterdam Ultra-high field adult lifespan database (AHEAD): A freely available multimodal 7 Tesla submillimeter magnetic resonance imaging database. *NeuroImage*, *221*, 117200. https://doi.org/10.1016/j.neuroimage.2020.117200

Chepkoech, J.-L., Walhovd, K. B., Grydeland, H., & Fjell, A. M. (2016). Effects of change in FreeSurfer version on classification accuracy of patients with Alzheimer’s disease and mild cognitive impairment. *Human Brain Mapping*, *37*(5), 1831–1841. https://doi.org/10.1002/hbm.23139

De Felice, F. G., Gonçalves, R. A., & Ferreira, S. T. (2022). Impaired insulin signalling and allostatic load in Alzheimer disease. *Nature Reviews Neuroscience*, *23*(4), 215–230. https://doi.org/10.1038/s41583-022-00558-9

de Moraes, F. H. P. (2021). *OpenData for the Independent morphological variables correlate to aging, Mild Cognitive Impairment, and Alzheimer’s Disease manuscript* [Data set]. Zenodo. https://doi.org/10.5281/zenodo.5750619

Dickerson, B. C., Fenstermacher, E., Salat, D. H., Wolk, D. A., Maguire, R. P., Desikan, R., Pacheco, J., Quinn, B. T., Van der Kouwe, A., Greve, D. N., Blacker, D., Albert, M. S., Killiany, R. J., & Fischl, B. (2008). Detection of cortical thickness correlates of cognitive performance: Reliability across MRI scan sessions, scanners, and field strengths. *NeuroImage*, *39*(1), 10–18. https://doi.org/10.1016/j.neuroimage.2007.08.042

Fischl, B. (2012). FreeSurfer. *NeuroImage*, *62*(2), 774–781. https://doi.org/10.1016/j.neuroimage.2012.01.021

Fortin, J.-P., Cullen, N., Sheline, Y. I., Taylor, W. D., Aselcioglu, I., Cook, P. A., Adams, P., Cooper, C., Fava, M., McGrath, P. J., McInnis, M., Phillips, M. L., Trivedi, M. H., Weissman, M. M., & Shinohara, R. T. (2018). Harmonization of cortical thickness measurements across scanners and sites. *NeuroImage*, *167*, 104–120. https://doi.org/10.1016/j.neuroimage.2017.11.024

Frisoni, G. B., Fox, N. C., Jack, C. R., Scheltens, P., & Thompson, P. M. (2010). The clinical use of structural MRI in Alzheimer disease. *Nature Reviews. Neurology*, *6*(2), 67–77. https://doi.org/10.1038/nrneurol.2009.215

Frozza, R. L., Lourenco, M. V., & De Felice, F. G. (2018). Challenges for Alzheimer’s Disease Therapy: Insights from Novel Mechanisms Beyond Memory Defects. *Frontiers in Neuroscience*, *12*. https://doi.org/10.3389/fnins.2018.00037

Gonçalves, R. A., Sudo, F. K., Lourenco, M. V., Drummond, C., Assunção, N., Vanderborght, B., Ferreira, D. D. P., Ribeiro, F. C., Pamplona, F. A., Tovar-Moll, F., Mattos, P., Ferreira, S. T., & De Felice, F. G. (2022). Cerebrospinal fluid irisin and lipoxin A4 are reduced in elderly Brazilian individuals with depression: Insight into shared mechanisms between depression and dementia. *Alzheimer’s & Dementia*, *n/a*, 1–10. https://doi.org/10.1002/alz.12893

Gronenschild, E. H. B. M., Habets, P., Jacobs, H. I. L., Mengelers, R., Rozendaal, N., Os, J. van, & Marcelis, M. (2012). The Effects of FreeSurfer Version, Workstation Type, and Macintosh Operating System Version on Anatomical Volume and Cortical Thickness Measurements. *PLOS ONE*, *7*(6), e38234. https://doi.org/10.1371/journal.pone.0038234

Heinen, R., Bouvy, W. H., Mendrik, A. M., Viergever, M. A., Biessels, G. J., & de Bresser, J. (2016). Robustness of Automated Methods for Brain Volume Measurements across Different MRI Field Strengths. *PLoS ONE*, *11*(10). https://doi.org/10.1371/journal.pone.0165719

Kim, J. P., Seo, S. W., Shin, H. Y., Ye, B. S., Yang, J.-J., Kim, C., Kang, M., Jeon, S., Kim, H. J., Cho, H., Kim, J.-H., Lee, J.-M., Kim, S. T., Na, D. L., & Guallar, E. (2015). Effects of education on aging-related cortical thinning among cognitively normal individuals. *Neurology*, *85*(9), 806. https://doi.org/10.1212/WNL.0000000000001884

Liu, Y., Julkunen, V., Paajanen, T., Westman, E., Wahlund, L.-O., Aitken, A., Sobow, T., Mecocci, P., Tsolaki, M., Vellas, B., Muehlboeck, S., Spenger, C., Lovestone, S., Simmons, A., Soininen, H., & AddNeuroMed Consortium. (2012). Education increases reserve against Alzheimer’s disease—Evidence from structural MRI analysis. *Neuroradiology*, *54*(9), 929–938. https://doi.org/10.1007/s00234-012-1005-0

Pamplona, F. A., Vitória, G., Sudo, F. K., Ribeiro, F. C., Isaac, A. R., Moraes, C. A., Chauvet, M. G., Ledur, P. F., Karmirian, K., Ornelas, I. M., Leo, L. M., Paulsen, B., Coutinho, G., Drummond, C., Assunção, N., Vanderborght, B., Canetti, C. A., Castro-Faria-Neto, H. C., Mattos, P., … Tovar-Moll, F. (2022). Age-linked suppression of lipoxin A4 associates with cognitive deficits in mice and humans. *Translational Psychiatry*, *12*(1), Artigo 1. https://doi.org/10.1038/s41398-022-02208-1

Rosen, A. F. G., Roalf, D. R., Ruparel, K., Blake, J., Seelaus, K., Villa, L. P., Ciric, R., Cook, P. A., Davatzikos, C., Elliott, M. A., de La Garza, A. G., Gennatas, E. D., Quarmley, M., Schmitt, J. E., Shinohara, R. T., Tisdall, M. D., Craddock, R. C., Gur, R. E., Gur, R. C., & Satterthwaite, T. D. (2018). Quantitative Assessment of Structural Image Quality. *NeuroImage*, *169*, 407–418. https://doi.org/10.1016/j.neuroimage.2017.12.059

Rutherford, S., Kia, S. M., Wolfers, T., Fraza, C., Zabihi, M., Dinga, R., Berthet, P., Worker, A., Verdi, S., Ruhe, H. G., Beckmann, C. F., & Marquand, A. F. (2022). The normative modeling framework for computational psychiatry. *Nature Protocols*, *17*(7), Artigo 7. https://doi.org/10.1038/s41596-022-00696-5

Snoek, L., van der Miesen, M. M., Beemsterboer, T., van der Leij, A., Eigenhuis, A., & Steven Scholte, H. (2021). The Amsterdam Open MRI Collection, a set of multimodal MRI datasets for individual difference analyses. *Scientific Data*, *8*(1), 85. https://doi.org/10.1038/s41597-021-00870-6

Stern, Y. (2012). Cognitive reserve in ageing and Alzheimer’s disease. *The Lancet Neurology*, *11*(11), 1006–1012. https://doi.org/10.1016/S1474-4422(12)70191-6

Wang, Y., Ludwig, T., & Mota, B. (2019). *Analysis pipeline to extract cortical morphology measures from Freesurfer for cortical folding analysis* (2.0). Zenodo. https://doi.org/10.5281/zenodo.3608675

Wang, Y., Necus, J., Kaiser, M., & Mota, B. (2016). Universality in human cortical folding in health and disease. *Proceedings of the National Academy of Sciences*, *113*(45), 12820–12825. https://doi.org/10.1073/pnas.1610175113

Whittington, R. A., Planel, E., & Terrando, N. (2017). Impaired Resolution of Inflammation in Alzheimer’s Disease: A Review. *Frontiers in Immunology*, *8*. https://doi.org/10.3389/fimmu.2017.01464

1. Here are two threads in the FreeSurfer forum regarding the Euler number <https://www.mail-archive.com/freesurfer@nmr.mgh.harvard.edu/msg58748.html> and <https://www.mail-archive.com/freesurfer@nmr.mgh.harvard.edu/msg67542.html>. [↑](#footnote-ref-2)
2. ADNI data used in the preparation of this article were obtained from the Alzheimer’s Disease Neuroimaging Initiative (ADNI) database ([adni.loni.usc.edu](file:///D:\idor\apresentações\Artigos\Submissao%20Brain%20-%20Cortical%20Folding%20AD%20and%20Aging\adni.loni.usc.edu)). As such, the investigators within the ADNI contributed to the design and implementation of ADNI and/or provided data but did not participate in the analysis or writing of this report. A complete listing of ADNI investigators can be found at: <http://adni.loni.usc.edu/wp-content/uploads/how_to_apply/ADNI_Acknowledgement_List.pdf>. [↑](#footnote-ref-3)
